# The future of the future foods: understandings from the past towards SDG-2

**DOI:** 10.1038/s41538-025-00484-x

**Published:** 2025-07-12

**Authors:** Mehvish Habib, Sakshi Singh, Shumaila Jan, Kulsum Jan, Khalid Bashir

**Affiliations:** 1 Department of Food Technology, Jamia Hamdard, New Delhi, India; 2 Department of Food Science & Technology, NIFTEM-K, Sonipat, India

**Keywords:** Nutrition, Science, technology and society

## Abstract

Food security faces growing challenges due to population growth, resource limitations, economic pressures, and industrialization-induced lifestyle changes. Traditional food systems struggle to adapt, necessitating innovative solutions and sustainable practices to meet future food demands. This review article explores emerging food system models and alternative food sources, including edible insects, seaweeds, plant-based and lab-cultured meats, underutilized crops, hydroponics, and next-generation fish farming. It highlights the role of food processing technologies such as blockchain, biotechnology, and robotics in enhancing sustainability, reducing waste, and improving food system efficiency. Consumer acceptance of engineered and fortified foods emerges as a critical factor in driving these innovations. The review also emphasizes the need for a transformative approach to food production, incorporating innovative technologies and sustainable practices to ensure food security by 2050. A coordinated effort to integrate alternate food sources and advanced processing methods will be vital for achieving a secure and sustainable global food future.

## Introduction

**Fig. 1:**
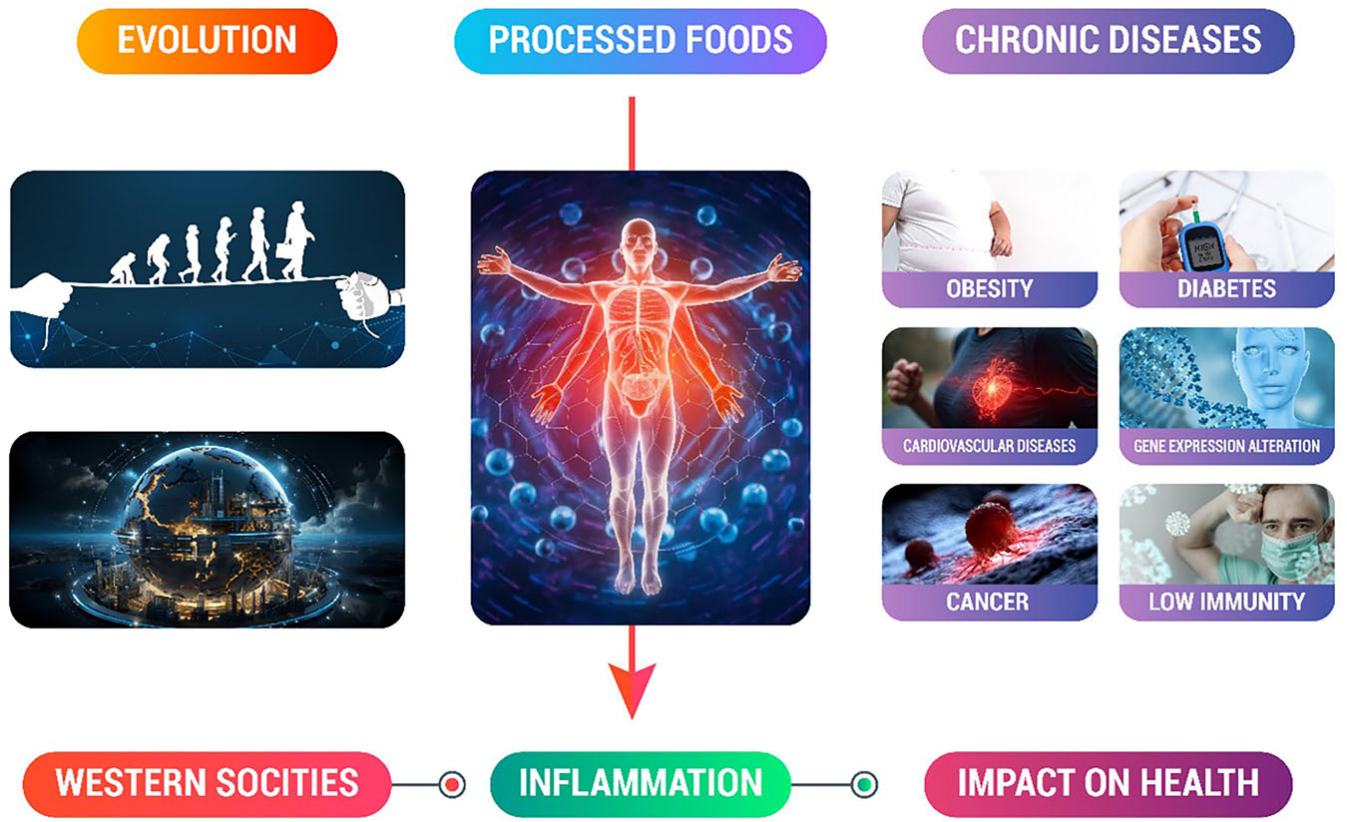
Description of human evolution and the changes in eating patterns caused by increased industrialization and the marketing of processed foods^5^.

Ensuring an adequate food supply for humanity has been a persistent challenge throughout history. The future of food is being shaped by evolving lifestyles, industrialization, and advancements in technology. Due to population growth and resource scarcity, food security is more important than ever. Many variables endanger global food systems, including population growth, climate change, conflicts, geopolitical concerns, pandemics, and consumer preferences. The global population is expected to exceed 9.7 billion by 2050, requiring resource management and production to provide safe, healthy, and sustainable food^1^. As global food consumption rises and the world urbanizes, environmental impacts and agriculture proportions rise, necessitating sustainable and resilient food systems. The future of food is a hot topic among experts and the public amid current concerns and opportunities. Therefore, marginal area yields must be increased, crop production raised, diet quality modified, and waste minimized under reduced environmental degradation^2^. To meet these expectations and promote the consumption of budget-friendly products, a transformation is required to align with the global shift from fossil fuels to bio-based alternatives, achieve climate neutrality, adopt advanced food logistics technologies, and enhance bioresource valorization. Policymakers must integrate urban horticulture, sky farming, smart farming, Information Technology (IT), and the ‘blue bioeconomy’ from aquaculture and polyculture with agroecological principles. They should also address food safety, digital traceability, efficient food distribution, livestock production in mixed systems, One Health, and short food supply chains. Alternative eating methods like plant-based and cultured meat should be pushed. With the advent of agriculture, novel staples emerged that were unfamiliar to the hominin genome. Additionally, food processing introduced new combinations of nutrients and foods. To understand these transformations, it is essential to consider both the nutrients and foods available to pre-agricultural hominins and those introduced through agriculture, industrialization, and technological advancements^3^. The transition from diverse plant-based foods to carbohydrate-rich sources with low fiber content, such as tubers, cereals, refined sugars, and highly processed, energy-dense foods, has rapidly occurred over the past few thousand years of human evolution due to farming, industrialization, and technological advancements. Early human evolutionary genetics differ significantly from modern lifestyles. Before the advent of agriculture and animal domestication, hominins relied solely on paleo-olotechnic diets composed of wild plants and animals. Micronutrient content changed with domestication of food crops and animals and technological improvement following industrialization. Pre-agricultural humans had little access to additional sugars and fats such as dairy products, cereals, refined sugars, vegetable oils, and alcohol, which contributes 72.1% of US energy intake. In addition to eating more processed grains, the average consumer now eats more cookies, cakes, bakery products, breakfast cereals, and other snacks, deviating further from traditional diets^4^. Human evolution has undergone major dietary transformations from paleolithic diets with total or mostly unpreserved foods to the agricultural revolution with grain products and the industrial revolution with globalization and marketing of convenience foods have changed eating habits by promoting calorie-dense, micronutrient-poor diets^5^. “Chronic inflammation causes obesity, diabetes, cardiovascular disease, and autoimmune illness. Only nutrient-dense diets that fight inflammation and other disorders can solve these issues (Fig. 1).”

**Fig. 2:**
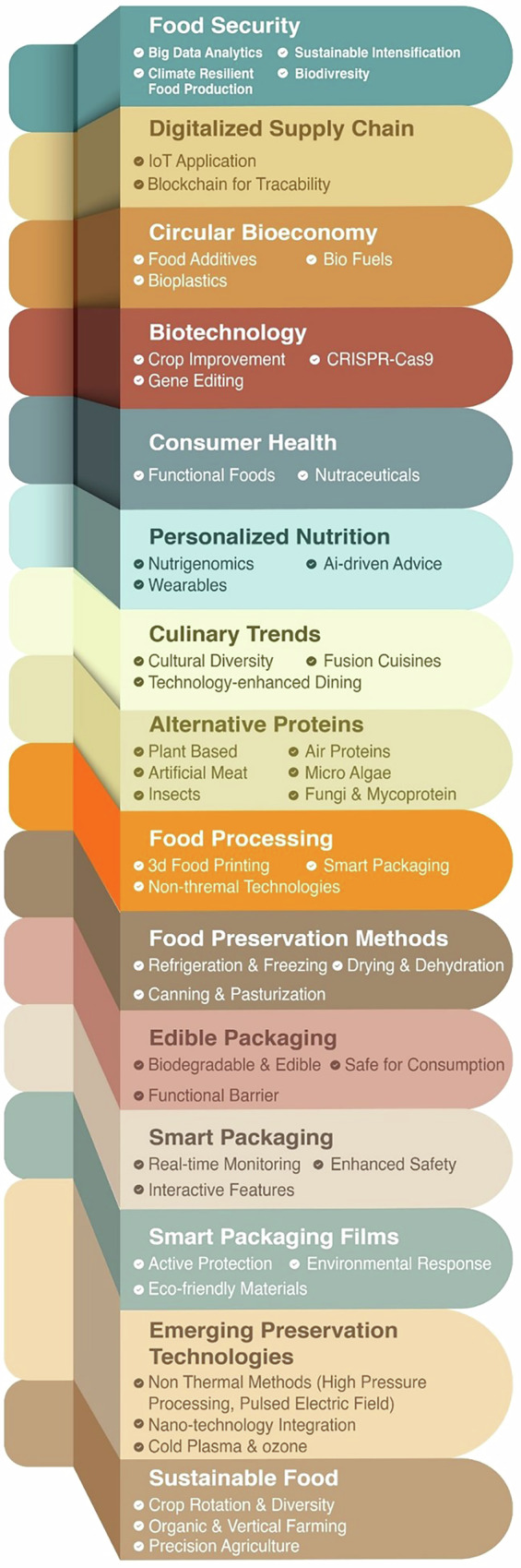
Illustration of the dimensions that constitute the future of food^6^.

The specific scientific objectives—such as transitioning to healthier diets and embracing sustainable consumption (which involves reducing red meat and sugar)—also include the production of nutritious and functional foods. Furthermore, the development of bioactive-rich foods and the customization of dietary choices through personalized diets and nutrigenomics, alongside the impact of cultural diversity on culinary innovations, are crucial for steering this transformation. However, other possible breakthroughs exist; for instance, advancements in crops using Clustered Regularly Interspaced Short Palindromic Repeats and CRISPR-associated protein 9 (CRISPR-Cas9) gene editing may raise concerns about safety and public perception^6^. Because of this, consumer preferences should not be underestimated, but rather seen as a vital framework for the creation of future food products as presented in Fig. 2

Modern lifestyles and rapid urbanization have significantly altered dietary habits, leading to a surge in demand for convenience foods, fortified products, and ready-to-eat meals. Although, this shift as served the modern needs, also brought change in the form of resource scarcity, environmental pressure, and questions regarding nutritional value^7^. Solving these challenges means finding a new way of organizing foods to feed people that is sustainable and may not rely heavily on the current uniform food production system that is vulnerable to climate shocks^8^. A sustainable food ecosystem can be achieved through a system incorporating edible insects, seaweeds, plant-based and cultured meats, underutilized crops, hydroponics, biotechnology, robotics, and blockchain technology^9^. This review aims to explore emerging trends and challenges shaping the future of global food security, examine the role of innovation in addressing food security issues, and analyze strategies for resource mobilization to achieve food security goals for the global population.

## Impact of lifestyle and industrialization on food habits

Integration of food production through industrialization has greatly influenced food consumption patterns and food chains (amending all cycles in the food chain). Rooted in modernization ideologies, it emphasized efficiency, standardization and mass production, often driven by business interests and technological advancements^10^. Key developments, such as appertization and innovations in organic chemistry, transformed food preservation and safety standards (while simultaneously reducing reliance on sensory evaluations). However, these changes encountered opposition from social movements advocating for natural and holistic approaches to nutrition. This opposition continues to influence contemporary ideologies, such as veganism and anti-speciesism, although the debate remains complex.

**Fig. 3:**
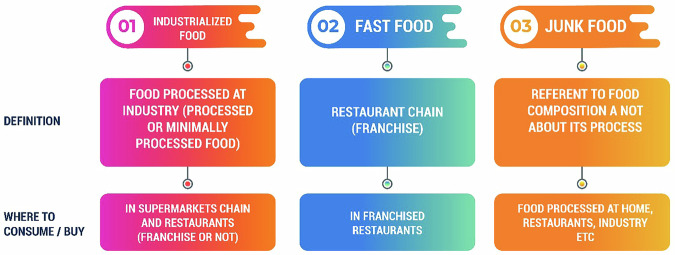
Fast food, junk food, and industrialized food—definitions and differences^201^.

More than one-third of global dietary guidelines recommend avoiding fast foods, yet there is often confusion between fast food, industrialized food, and junk food. Industrialized food refers to products processed on a large scale using industrial equipment, which are distributed through retail outlets and restaurants^11^. Fast food, on the other hand, is characterized by its operational model, which often employs industrialized products but focuses on rapid preparation and service, akin to Fordism. Junk food is defined by its low nutritional quality, regardless of whether it is prepared in industries, homes, or restaurants. To clarify: junk food is determined by its nutrient composition, fast food is tied to a restaurant’s style of operation, and industrialized food pertains to mass production methods as shown in Fig. 3.

The mid-20th century (a period marked by significant industrialization) notably altered dietary patterns, primarily due to the rising popularity of convenience foods and ready-to-eat products. This shift was largely influenced by changing social dynamics, particularly the increasing participation of women in the workforce. As a result, the consumption of calorie-dense, processed foods surged; consequently, this led to a notable rise in obesity rates and the prevalence of noncommunicable diseases. Furthermore, industrial food practices introduced various environmental and ethical dilemmas. These include (but are not limited to) pollution, deforestation and growing concerns regarding animal welfare, as well as cultural homogenization. Criticism directed at industrialized food systems often centers on the health risks associated with processed products, ethical issues related to exploitation and market manipulation and environmental repercussions such as biodiversity loss and overfishing. Although some objections may seem philosophical or sensationalized, it is essential to recognize that many are substantiated by scientific evidence linking processed foods to adverse health outcomes and the detrimental effects of industrial agriculture on the environment. These ongoing challenges (which are increasingly relevant) continue to influence conversations surrounding the sustainability, safety and ethics of our food systems^12^.

Technological advancements in food Industry have significantly transformed food systems, revolutionizing how food is produced, preserved, distributed, and consumed. Innovations such as mechanized agriculture, enhanced transportation networks, and advanced preservation techniques have improved efficiency, extended shelf life, and expanded access to diverse food options. These developments have streamlined food processing and distribution, ensuring consistent quality and availability while addressing the growing demands of urban and global populations as outlines in Table 1.

**Table 1:** Technological Advancements and Social Shifts in Food Systems^183,184^

Technological Advancements in Food Systems	Key Developments
Mechanization of Agriculture	Introduction of tools like plows, reapers, and threshers increased efficiency and reduced manual labor.
Enhanced Transportation Networks	Railways and refrigerated rail cars expanded perishable goods’ distribution to urban and remote areas.
Advances in Preservation Technologies	Innovations like canning, pasteurization, and refrigeration extended food shelf life and minimized spoilage.
Mass Production Techniques	Streamlined food processing ensured consistent quality and efficiency, e.g., products like Heinz ketchup and Quaker Oats.

Social Shifts Influencing Dietary Habits	Key Developments
Urbanization and Globalization	Increased demand for convenient, affordable food options; growth of public markets and grocery stores.
Changing Consumer Preferences	Higher disposable incomes diversified food choices, leading to the popularity of cereals, canned soups, and global cuisines.
Changing Gender Roles	Increased workforce participation by women boosted demand for pre-prepared foods like boxed cake mixes and frozen dinners.

Impact of Processed Foods on Public Health	Key Developments
Packaged and Convenience Foods	Offered long shelf lives and easy preparation but introduced preservatives and unhealthy ingredients.
Rise in Diet-Related Diseases	High levels of sugar, salt, and unhealthy fats linked to obesity, diabetes, and heart disease.
Decline in Home Cooking	Homogenized culinary traditions and diminished regional food cultures.

Environmental and Ethical Challenges of Industrialized Food Systems	Key Developments
Intensive Farming Practices	Monoculture cropping caused biodiversity loss, soil degradation, and water pollution.
Factory Farming	Raised concerns about animal welfare, antibiotic resistance, and disease outbreaks.
Environmental Impact of Waste	Highlighted inefficiencies through food waste and non-biodegradable packaging.
Exploitative Labor Practices	Underscored ethical concerns, particularly in migrant and seasonal labor.

*Source: Sproesser et al.,2017; Knorr and Watzke, 2019; Sproesser et al., 2022; Espeitx et al., 2017.*

## Food system models

**Fig. 4:**
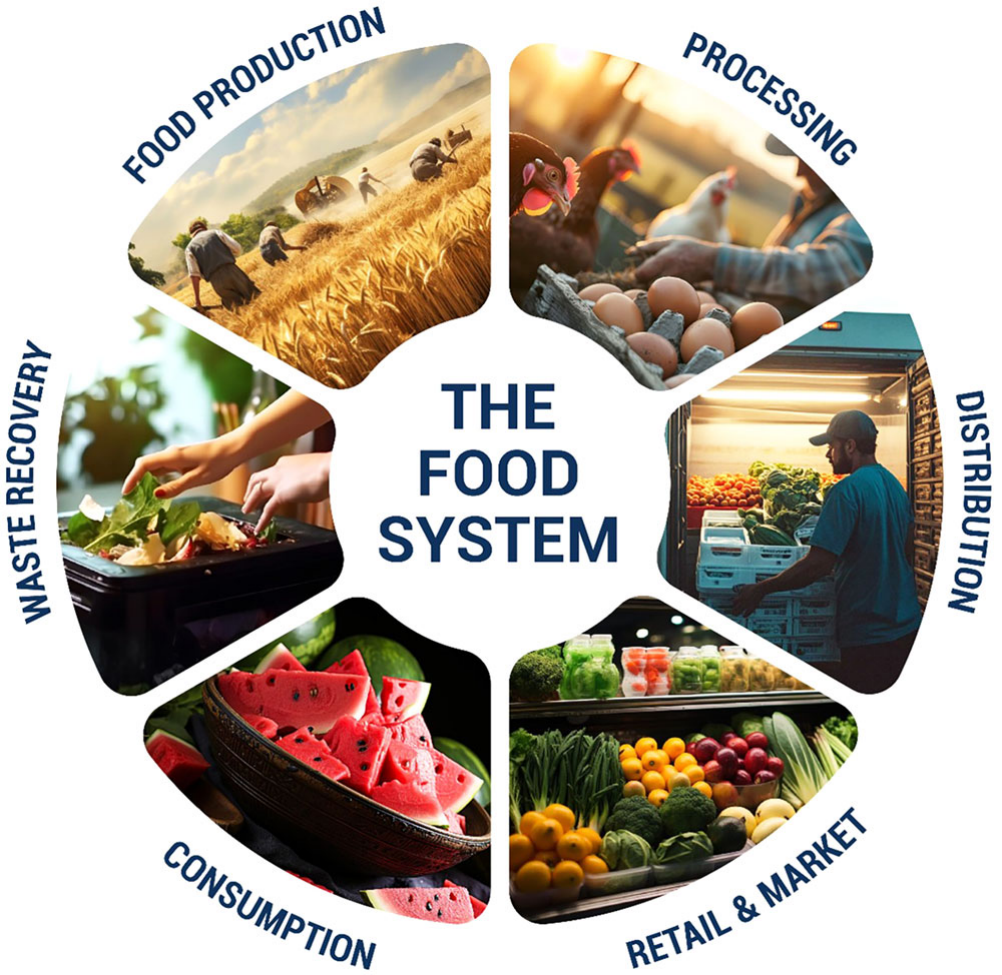
An Overview of Food System^185^.

The food system in the United States is vast and intricate, involving numerous stakeholders across various sectors. The Food Policy Councils are indispensable tools to facilitate the creation of strong food chains as an assembly of stakeholders and community leaders seeks to solve food system issues. These councils, led by either governments or, in some instances, NGOs, unite experts in agriculture, health, and economics to assess food systems, provide recommendations, and enhance resource sharing. Besides policy advocacy, they also conduct advocacy-education like how Kansas has helped increase access to SNAP or North Carolina’s Healthy Corner Stores Act. Similarly, policies governing land use for food production are inclusive of tax exemptions and easements for agricultural uses of land in order to promote rural vitality and conscious utilization of natural resources for agriculture^13^. The work of implementing these models depends on the support of community, trust and proper exploitation of resources including grants which Michigan State University Center for Regional Food Systems has pointed out in its collective impact strategies. Unlike models that focus on isolated components such as production or processing, this whole-system approach aims to address food access challenges comprehensively. Food access issues typically span multiple areas within the food system, and tackling just one part often fails to resolve the underlying problems. By engaging voices from all facets of the food system, this model adopts a holistic strategy to identify and address these challenges effectively as shown in Fig. 4.

The global food system has experienced substantial transformations over time—shaped by changes in technology, policy, and environmental pressures. From traditional agricultural methods that emphasized local production and sustainability to modern industrial food systems that prioritize large-scale, mechanized production, food systems have continuously evolved to meet the demands of growing populations. As we confront pressing challenges like climate change, resource scarcity and population growth, the future of food systems is shifting toward sustainability and resilience. This transition focuses on integrating innovative practices and technologies that can ensure food security while minimizing environmental impact^14^; however, achieving these goals is complex. Although efforts are underway, the path forward is fraught with obstacles, which requires a concerted effort from multiple stakeholders.

### Traditional agricultural practices

Traditional food systems were deeply rooted in localized agricultural practices that emphasized sustainability and resilience. These systems were characterized by:

- ***Subsistence farming***: Small-scale, family-oriented farming focused on local food production.
- ***Biodiversity***: The use of diverse crops and livestock, promoting ecological balance and resilience.
- ***Minimal processing***: Foods were typically consumed fresh or preserved using natural methods like drying and fermentation.

**Fig. 5:**
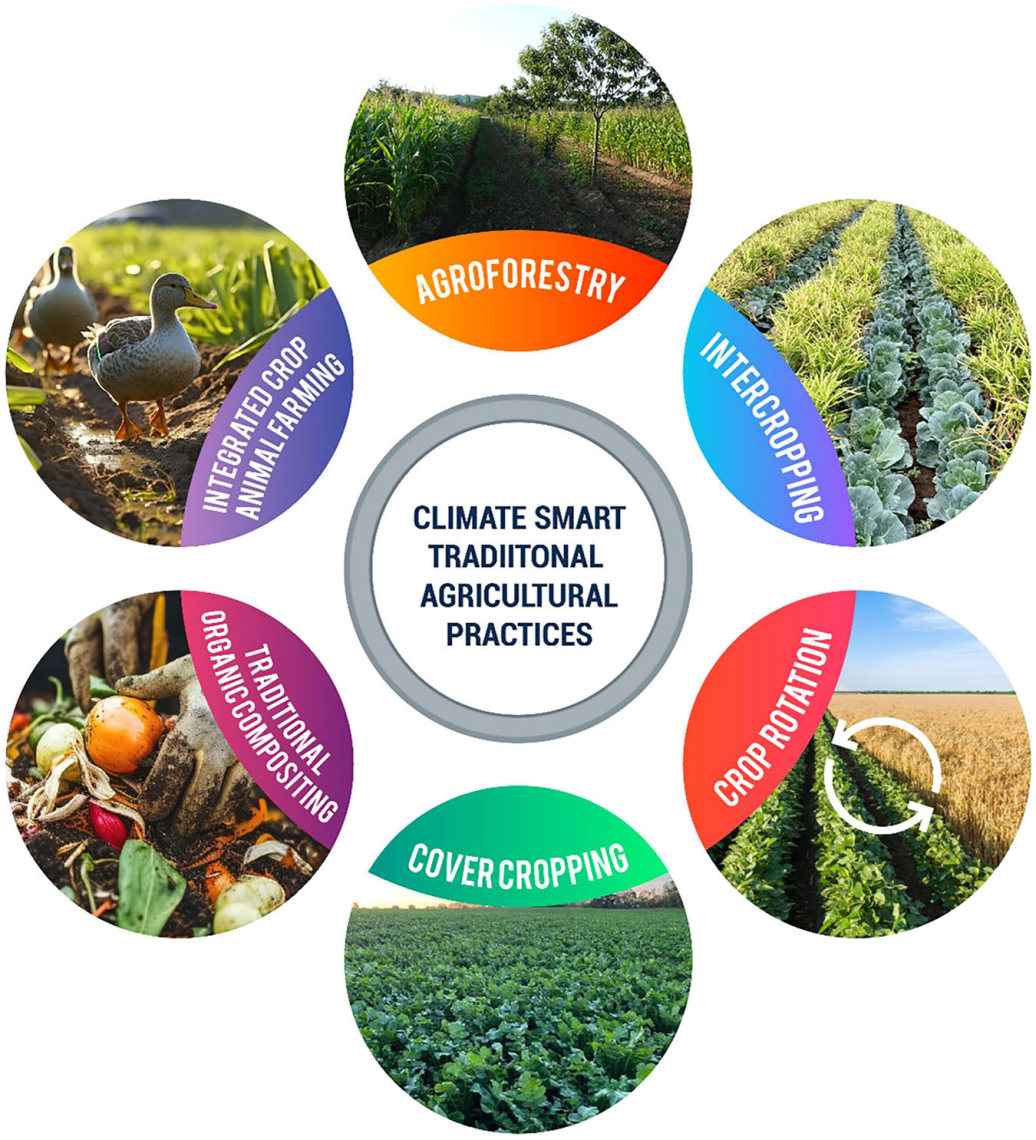
Climate-smart traditional agricultural practices^15^.

Traditional knowledge has been used to manage environmental services for millennia in agriculture, climate conditions, water and hydrologic systems, and human nutrition. Based on this understanding, farming and husbandry have shaped agricultural science and fed 1.9–2.2 billion people. Smallholder farmers, who work on 12% of the world’s agricultural land, are crucial in maintaining existing methods and adapting to changing climates utilizing local knowledge. Average farmers make up 80% of Sub-Saharan Africa’s farms and are more productive than large-scale farming. The Western Ghats in India and the Hani rice terraces in China demonstrate that conventional agricultural practices can increase efficiency by sustaining biological variety to maturity. Increasing monoculture production has been shown to harm agricultural biodiversity. Small-scale farming, or agro-ecological systems, are becoming development remedies to deadly industrial agricultural practices as climate change influences progress. Tree planting in farmer’s fields, cultivating two or more crops on one field, organic compost, crop rotation, and cover crops absorb carbon and minimize greenhouse gasses. Agroforestry systems reduce nitrous oxide and methane emissions and increase carbon stocks, while intercropping improves food security and soil organic carbon (Fig. 5). Table 2 provides a detailed overview of climate-smart traditional agricultural practices and their roles in mitigating climate change. While traditional systems have supported sustainability through minimal environmental impact and reliance on natural cycles, challenges in scalability and food security during adverse conditions have become evident. Moving forward, there is potential to integrate traditional practices with modern agricultural technologies to create more resilient and sustainable food systems^15^.

**Table 2:** Climate-smart traditional agricultural practices and their role in climate change mitigation

Traditional Practices	Location	Brief Description	Climate Change Mitigation	References
Agroforestry	West and Central Africa	Using traditional cocoa agroforestry techniques to use resources and land sustainably	Improves carbon sequestration and reduces emissions of N_2_O and CH_4_	^185^
Intercropping	Latin America	Intercropping beans with potatoes, maize, and other crops	Increase carbon sequestration	^15^
Crop Rotation	Southern Spain	Switching between sunflower and durum wheat	Increases soil nitrogen and carbon sequestration.	^16^
Cover Cropping	Paraguay	Utilizing mucuna leaves with gray seeds as cover crops	Increases the pool of soil organic carbon (SOC)	^186^
Traditional Organic Composting	India	Utilization of composted farmyard manure (FYM) in villages in the Indian Himalayas	Improves the sequestration of carbon by increasing soil organic matter (SOM).	^12^
Integrated Crop-Animal Farming	China	Duck-rice-fish culture system	Reduces external nitrogen fertilizers, lowers N_2_O emissions	^183^

### Modern industrial food systems

Modern food production and distribution is organized by globalization, mechanization, and mass manufacturing into a complicated system. Industrial agriculture, especially with tractors and other farm technology and chemicals, permits large-scale agricultural production. Since food is produced in one country or region and provided in others, global supply chains increase carbon food-miles but improve consumer food availability. Convenience is also important, and new trends in processed and packaged food consumption affect worldwide diets.

Food system includes food production, inputs, handlers/processors, equipment, structures, and food itself from production to consumption and disposal. It also shows how these activities affect nutrition, food security, socioeconomics, and the environment. The food system relies on livestock producers, milking facilities, processing firms, shipping companies, merchants, marketing agencies, and suppliers in the milk industry. This intricate web conforms to different regulatory agencies that ensure food safety and quality^16^.

**Fig. 6:**
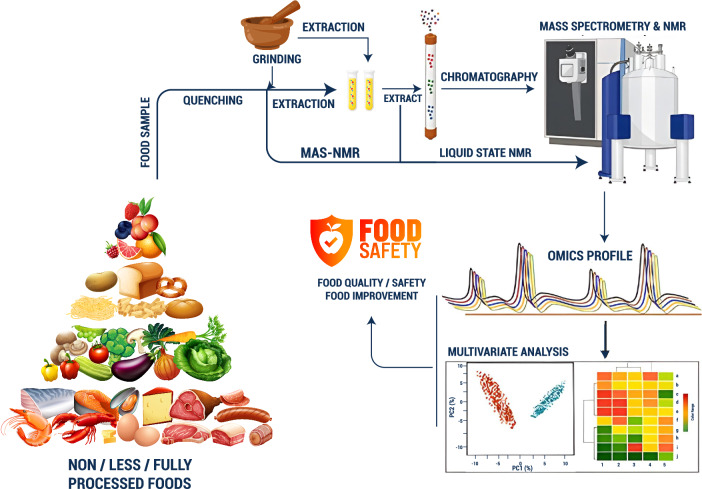
Regulating Modern Food Systems: Challenges and Opportunities^13^.

Different bodies govern today’s food systems to protect consumer interests. The above agencies ensure food manufacturers, packagers, and conveyors meet rigorous requirements. The FDA and USDA oversee food safety in the US for items with low infectious agent risk and high-risk control agents, such as the EPA. Food safety covers production, processing, and recalling tainted goods, but the FDA regulates all foods, domestic and foreign. The USDA oversees health risks related to meat, poultry, and processed egg products, as well as agriculture and food aid, while the EPA, through the FQPA, ensures the safety of food pesticides for children^14^. The USDA conducts pesticide residue tests, the FDA verifies food safety claims, and the EPA oversees pesticide safety. They maintain the safety of national and international food supply chains. Modern food systems depend on food preservation and processing for safety, quality, and shelf life. Freezing, salting, smoking, and irradiation reduce spoilage and foodborne illness. However, excessive sodium consumption from salting or small nutritional losses from freezing raise health and nutrition problems. Food processing enhances convenience and flavor, but ultra-processed meals contain artificial additives that deplete nutrients and pose health hazards. Preservatives and flavor enhancers are required for consistency and safety, but they may offer long-term health risks. Implementing strong laws and doing continual research is necessary to weigh the pros and cons of these practises. Figure 6 shows that this method makes current food systems safe, sustainable, and nutritionally beneficial.

### Future: sustainable and resilient food systems

To feed the global population in the year 2050 which is estimated to be 10 billion, the food production is going to increase by 70%, however traditional methods that has been in practice of simply increasing production is no longer sustainable. The new food regimes require changes in consumption patterns, decline in animal-based diets, and development of climate-smart agricultural systems. The key strategies are: reducing post-harvest losses, improving the processing of raw materials, and adopting sustainable practices such as organic farming, agroforestry, and crop rotation. Recent advancements, such as vertical farming, precision agriculture, and the integration of artificial intelligence, the Internet of Things, and cyber-physical systems, are expected to improve farming efficiency and optimize resource utilization in a more sustainable way. It shows that these technologies will help make agriculture sustainable because they enhance crop observation, resource utilization, and minimized environmental effects^17^. It will therefore be important to forge partnerships throughout the entire food system and promote policy measures that will ensure future proof against climate change and management of limited resources.

## Land use and food use by 2050

By the year 2050, the expanding global population (along with) urbanization and the strain on natural resources will considerably influence land use and food demands. With an anticipated 70% rise in global food production, additional agricultural land will be necessary; however, this will be offset by the implementation of sustainable practices like intensified farming and precision agriculture. Although a transition to plant-based diets might lessen the requirement for grazing land, it could potentially liberate space for alternative crops^18^.

- Increased Food Demand: A significant 70% rise in food production will necessitate an expansion of agricultural land.
- Land Intensification: Advanced farming techniques—such as precision agriculture—will maximize yields on existing plots.
- Dietary Shifts: More individuals adopting plant-based diets may, however, reduce the demand for grazing land.
- Climate Change Impact: Adaptations, including drought-resistant crops, will be essential for future resilience.
- Sustainable Practices: Approaches like agroforestry, cover cropping and reduced tillage will help minimize environmental impact.

**Fig. 7:**
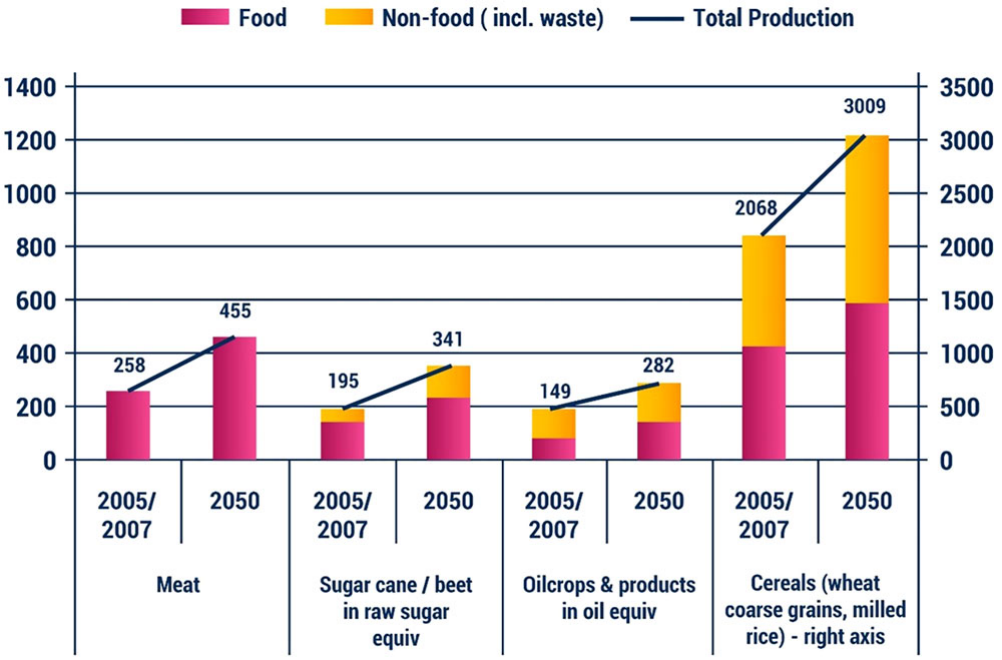
World production and use, major products (million tonnes)^202^.

Projections indicate two contrasting scenarios for food consumption by 2050: the Agrimonde GO scenario, which predicts higher food availability driven by economic growth and the Agrimonde 1 scenario, which focuses on health, equity and sustainability^19^. This latter scenario anticipates a more modest increase in food availability, along with a shift towards plant-based foods. Both scenarios suggest a decrease in animal product consumption, with plant-based and aquatic foods becoming more prominent; although, this transition may not be straightforward as shown in Fig. 7.

Due to resource restrictions and public health challenges, food consumption hypotheses predict that diets will change to meet sustainable development goals. This means that consumers, producers, and politicians must consider the global and local health and environmental impacts of food production and consumption. These hypotheses address four main issues: First, the Food Security Gap is the difference between food supply and security needs (affluent nations exceed the 3000-kcal threshold), and a lot of food is wasted owing to eating habits. Second, Equity in Food Access entails worldwide convergence in average food availability, addressing regional inequalities. Third, according to the Food/Health Nexus, maintaining a daily intake of 3000 kcal per capita could reduce undernutrition in emerging nations and obesity. To achieve these aims, multiple sectors must work together. Meeting the needs of 9 billion people by 2050 would put a strain on natural resources. Given the increased use of animal products, which require plant calories, water, and energy and emit greenhouse gases, this is especially true. Ruminant cultivation in areas unsuitable for conventional crops can optimize land use and enhance carbon storage; however, while challenging, these benefits should not be overlooked^20^.

## Consumer acceptance of engineered foods

Consumer acceptance of genetically modified (GM) foods is shaped by a multitude of factors: organizational perceptions of benefits associated with the decision, perceived risks for undertaking the decision and perceived information available^21^. With this understanding, GM foods are often viewed favorably, particularly in developing countries, due to their potential to enhance food security, improve nutrition, and stimulate economic growth. For example, bioengineered foods like golden rice used to address the Vitamin A shortage get positive reactions in countries like the Philippines where malnutrition is still a major problemSimilarly, in China and Argentina, crops like Bt cotton and RR soybeans have boosted productivity and competitiveness, contributing to the acceptance of GM foods even in developed countries^22^. Thus, it can be mentioned that positive perception occurs due to the fact that in these regions, the nutritional and economical advantages of such foods exceed the perceived threats observed.

**Fig. 8:**
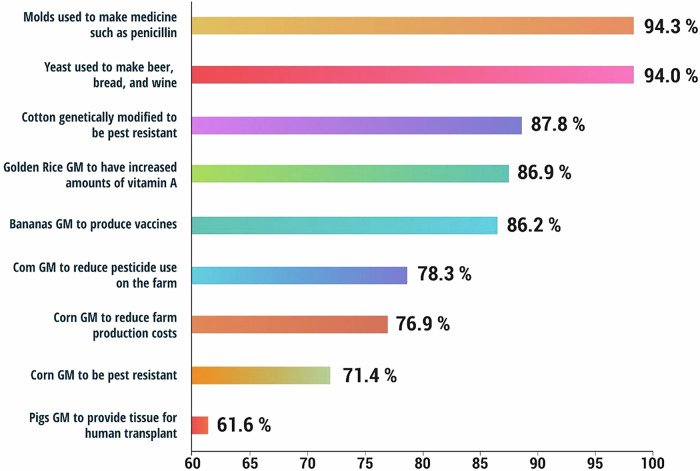
Consumer acceptance levels of GM foods, with traditional food practices like yeast and mold use in production being the most widely accepted^24^.

Consumer attitudes, however, in developed countries (Europe and Japan, etc.) differ greatly and are much more cautious. This skepticism stems from issues on effects on health and impacts on the environment in case of an accident. Previous food scandals have eroded public confidence in government regulators; therefore, increasing alertness has become the norm. Also, European consumers and Japanese conventional food systems have more concern regarding effects of genetically modified (GM) foods than any other group^23^. Thus, flows regulating the utilization and labeling of GM foods have been implemented in these areas. However, in the United States where food availability is most of the time reliable, consumers’ acceptance level is relatively high. This is the case, mostly because there is lesser coverage from the media, and high amount of confidence placed in the regulatory bodies, such as the FDA and EPA. Overall, despite seeming somewhat puzzling, the sentiment remains notably more positive than that of consumers in Europe or Japan. As indicated in the Fig. 8, there is low consumer acceptance of GM foods. Therefore, global acceptance based on the major drivers of acceptance was moderate, where most consumers rated Genetically Modified Foods as acceptable. As expected, non-GM food practices received the highest approval as evaluated by the respondents; most of the respondents preferred traditional food practices^24^. Users were supportive of the use of yeast in the production of beer and bread making and molds that are used in the production of medicine such as penicillin and regarded them as an acceptable methods of food production^25^.

Overall, the acceptance of GM foods among consumers is shaped by a complex set of factors, such as local food security needs, economic benefits, trust in regulations, and cultural attitudes^26^. Developing countries often adopt GM foods due to their practical advantages, while developed nations tend to be more cautious, emphasizing safety and environmental concerns. Despite regional differences, there is a growing demand for transparency—particularly in labeling—to empower consumers to make informed choices.

## Alternate food sources for a sustainable future

As the global population continues to grow, placing increasing pressure on the environment, ensuring sustainable food production has never been more critical. While modern conventional farming plays a key role in meeting today’s food demands, it faces numerous challenges such as land degradation, water scarcity, climate change, and rising food needs. These limitations highlight the importance of identifying alternative food sources as key elements for building a sustainable future. Table 3 summarizes sustainable sources (SS), innovative sources (IS), detailed innovations, their impact on sustainable diets, and environmental benefits when exploring alternative food sources for a more sustainable future.

**Table 3:** Alternate food sources and technological innovations for a sustainable future

Alternate Food Sources for a Sustainable Future	Sustainable Diet	Environment-Friendly Reasons	References
Edible Insects	High nutritional value, rich in protein, vitamins, and minerals.	Minimal land and water use, low greenhouse gas emissions.	^187^
Seaweeds	Rich in vitamins, minerals, and antioxidants, supporting diverse diets.	Requires no land, freshwater, or fertilizers; absorbs CO_2_.	^188^
Cultured Meat Technology (cell-based and 3D-printed plant-based meat)	Offers protein alternatives with potential for customization and reduced animal welfare concerns.	Requires no land, freshwater, or fertilizers; lower carbon footprint than conventional meat.	^189^
Plant-Based and Lab-Cultured Meat	Provides sustainable protein alternatives to traditional meat.	Low carbon footprint; reduces livestock farming and land use.	^190^
Gen Next Fish Farms	Sustainable seafood production to meet global demand.	Improves efficiency in aquaculture, reducing overfishing pressure.	^191^
Underutilized Crops	Revitalizing traditional crops for better nutrition.	Ecologically beneficial, adaptable to diverse climates, and drought-resistant.	^192^
Hydroponics	Urban farming solution for local food production.	Water-efficient, minimal land usage, reduces food miles.	^65^
Blockchain Technology	Improves food traceability, ensuring sustainable sourcing.	Enhances transparency, reduces waste, and supports ethical production.	^71^
Biotechnology in Food	Genetic engineering to enhance nutritional quality.	Reduces the need for chemical inputs, promotes environmental health.	^75^
Robotics in Food	Automation of food production for higher efficiency and scalability.	Reduces energy consumption, optimizes resource use, and minimizes waste.	^85^

### Edible insects

Entomophagy—defined as the consumption of insects—has gained significant attention as a sustainable alternative food source. In various regions of India, tribal communities have long integrated insects into their diets. For instance, in Nagaland, residents consume grasshoppers, crickets, red ants and mulberry silkworm larvae, favoring green larvae that are found on golmohar trees during the months of March and April. Similarly, communities in West Bengal and Odisha partake in red ant eggs, larvae and termites, with the latter collected during swarming seasons while red ants are harvested from plants. In Jharkhand, people from Pithra village are known to eat eggs from Demta, a type of red ant that inhabits trees. In other areas—such as Karnataka and Tamil Nadu—winged termites are consumed, whereas in Kerala, numerous species, including bees, ants and termites, comprise the local dietary landscape^27^. Furthermore, tribes in Arunachal Pradesh have been reported to consume over 80 species of local insects, which include silkworm caterpillars and pupae. In Meghalaya, the use of termites as a protein and mineral source has been noted; proponents claim that these nutritional values surpass those found in conventional vegetarian foods and meat. However, the practice continues to be met with both curiosity and scepticism^28^. In Assam, a variety of insects—such as red tree ants, crickets and honeybee larvae—are regularly consumed; the Ahom community, for instance, incorporates red tree ants during their Bohag Bihu festival. Insects like these are thought to provide health benefits because they offer protection against diseases, including scabies and malaria. However, some may find the idea of eating insects unappealing, although the nutritional advantages cannot be overlooked. This unique dietary practice reflects a cultural significance that goes beyond mere sustenance^29^. Insect consumption is not limited to India but is a global practice, particularly in tropical regions. Cultures across Africa, Asia, and Latin America consume insects as a source of protein. In areas facing food insecurity or economic instability, insects offer a protein-rich, low-maintenance dietary option^30^. Tropical locations have more insects, making insect gathering easier than temperate zones, where insects hibernate. In some Asian countries, ant eggs are a delicacy, even though ants, termites, and crickets are nutritious. This approach contributes to global food security by offering a sustainable, protein-rich alternative to conventional meats. In economically challenged areas, this is vital.

Edible insect nutritional profiles vary by species, habitat, metamorphic stage, and diet. Around 1900 edible bug species have been cataloged. The order *Coleoptera* (beetles) dominates, followed by *Lepidoptera* (butterflies), *Hymenoptera* (bees/wasps), and *Orthoptera*. Some species of these insects have more protein than meat and fish. Crickets have 61% protein, while termites have 35%^31^. However, grasshoppers are heavy in protein, with some species reaching 77% by dry weight. Insects provide essential amino acids, vitamins, and minerals such as iron, zinc, potassium, calcium, and magnesium, making them an excellent source of nutrients, unsaturated fatty acids, and antioxidants like carotenoids. Edible insects provide a long-term solution to protein deficiency, especially in underdeveloped nations where malnutrition is frequently compounded by a lack of access to conventional animal protein sources. These insects, especially pupae and larvae, are rich in amino acids and fatty acids, which are vital to human health. Their protein level is comparable to meat, but they are cheaper and easier to make. Since insects grow faster than chickens and cattle, they are a more efficient and accessible protein source. Edible insects are very easy to catch and prepare, making them a good dietary supplement for resource-poor people. Edible insects may help solve global protein shortages and food security difficulties, but consumer acceptance is still a problem. Bioactive substances and health advantages of edible insects are becoming more well recognized^32^. Bioactive compounds like β-carotene have been found in caterpillar species like *Imbrasia oyemensis*, *I. truncata*, and *I. epimethea*, with levels of 6.8 to 8.2 μg per 100 g of dry mass. Polyphenols and their metabolites, such as kaempferol-3-O-glucoside and kaempferol-3, 7-di-O-glucoside, are considered biotransformation products of plant-derived kaempferol. Other compounds found in edible insects include myricetin-3-O-rhamnoside, quercetin, and kaempferol. The mechanisms through which these compounds exert their effects remain unclear, requiring further research to fully understand their health benefits. While studies are ongoing, this field holds significant potential for nutritional exploration^31^.

Total edible insect sales are expected to reach $522 million by 2023. Foods made from insects are being developed to retain their appearance. Black army flies, yellow mealworms, silkworms, grasshoppers, and termites are popular insect-based animal feeds since they are cheaper than standard livestock feeds. These feeds cover a lot of animal production costs. Broiler diets can replace up to 4% of fish meal with insect meals like housefly-larvae meal without impacting carcass weight or feed efficiency^28^. In laboratory environments, artificial diets that are enriched with edible insects have shown numerous advantages when compared to natural plant-based diets for the rearing of species such as silkworms. These semi-synthetic diets (which incorporate ingredients like rice seed heads, wheat bran and chicken egg buster) are abundant in essential fatty acids. For instance, larvae that are fed a diet containing corn starch, vegetable oil and glucose exhibit superior nutritional profiles; this includes higher levels of protein and fat content. Insects such as the *Gonimbrasia belina* larvae have been observed to survive and flourish on diets primarily composed of corn stover, thus demonstrating the considerable potential of insect-based diets for sustainable food production. The nutritional composition of edible insects varies widely because essential amino acids like lysine, tryptophan and threonine are present in quantities that can effectively complement deficiencies found in traditional cereals and tubers. On average, insects contain between 10% and 70% fat in dry matter. However, larvae and pupae stages tend to have a higher fat content in comparison to adults. The distribution of fats in edible insects is also quite diverse, as triacylglycerols constitute approximately 80% of total fats while phospholipids account for a smaller fraction^30^. Beyond their nutritional value, edible insects present significant environmental advantages. They are (in fact) more eco-friendly than livestock, needing fewer natural resources—like water, land and energy. As global food production must rise to satisfy the demands of an expanding population, edible insects offer a sustainable and efficient alternative to conventional animal farming. Traditional methods significantly contribute to environmental degradation and greenhouse gas emissions; however, incorporating insects into diets could help address these challenges. While some may hesitate to adopt this alternative, the advantages are undeniable.

### Seaweeds

Seaweed is getting to be used as a good source of protein with least using up of land and water. Nowadays, the requirement for protein sources is constantly increasing internationally and especially for plant-based products, to which sea kale can be attributed as a complete source of protein since it contains all the necessary amino acids. Seaweed protein market may touch nearly 1 billion USD mark by 2032^33^. Seaweed farming supports environmental conservation as it requires no freshwater, fertilizers, or land clearing for cultivation. Instead, seaweed absorbs nutrients directly from seawater, making it a sustainable and eco-friendly practice. Also, it bears a climate mitigation function through holding carbon and supporting marine organisms. The European market share in the consumption of seaweed for food items in particular has risen by 147% in the period between 2011 and 2015 underlining its profile as the ‘super-food’^33^.

Seaweed is a broad category of macroalgae that has drawn a lot of interest as a renewable and natural source of valuable compounds with uses in a variety of industries. Packed with vital nutrients, biopolymers, and bioactive components, seaweed provides sustainable substitutes for traditional ingredients that support the circular economy. Because of its quick growth, capacity to absorb carbon dioxide, and low land and freshwater needs, it is a resource with enormous potential that is also environmentally beneficial^34^.

Biopolymers like agar, carrageenan, and alginate, which are abundant in seaweed, are utilized extensively in the food, medicine, and cosmetics sectors. In a variety of compositions, these natural polymers take the place of synthetic additives as stabilizers, emulsifiers, and gelling agents. Seaweed also provides vital elements that support human health and wellness, such as proteins, vitamins, minerals, and antioxidants. Its functional properties are further enhanced by the presence of bioactive components including polyphenols and sulfated polysaccharides, which makes it a valuable ingredient in functional meals and nutraceuticals. Seaweed-derived compounds have become more and more popular in the cosmetics sector due to its anti-aging, skin-protective, and hydrating qualities. Seaweed extracts, which are high in moisturizing compounds and antioxidants, are frequently used in skincare products to increase skin suppleness, lower inflammation, and shield the skin from environmental assaults. Seaweed is a sustainable substitute for synthetic and animal-derived compounds in personal care products because of its natural bioactive components, which aid in the formation of collagen^35^.

For sustainable animal feeding, especially in aquaculture, seaweed is essential. It is a nutrient-rich feed source that gives fish, shrimp, and crustaceans the critical omega-3 fatty acids, amino acids, and trace elements they need to thrive and stay healthy. Seaweed-based feeds provide a more environmentally friendly and sustainable substitute for traditional feed materials like fishmeal and soybean meal, which lessens the environmental impact of aquaculture. Furthermore, some seaweed species have bioactive substances that strengthen immunity and promote gut health in farmed aquatic species, lowering the need for antibiotics and improving sustainability in general^36^.

Seaweed has tremendous potential in sustainable agriculture as a soil conditioner and biofertilizer, in addition to its uses in food and feed. Fertilizers made from seaweed are abundant in vital elements like potassium, phosphorus, and nitrogen that improve soil fertility and encourage plant development. These organic fertilizers increase microbial activity, improve water retention, and fortify plant resistance to disease and drought. Seaweed extracts have also been demonstrated to promote crop yields and plant growth, providing a sustainable substitute for synthetic fertilizers that worsen soil conditions and contaminate water supplies^37^.

Aquaculture in particular, especially seaweed farming is increasingly being embraced as a more viable and sustainable form of husbandry as compared to most forms of conventional farming that can be very destructive to the environment. Currently the confined traditional soil-based farming practices that include deforestation, pest control by spraying chemicals, and excessive water rationing are some of the major culprits for the unchanged climate condition destruction of ecosystems. In addition, climate change is making these challenges worse by altering the weather patterns and raising the pest risk to food production. Seaweeds farming is another approach that not only is a better solution of this problem but is also suitable for the changing climate^38^. Seaweed farming can not only be considered as a supply source of food in the future but also has a positive effect on ocean acidification, as it absorbs the CO_2_ from the water and might also be used as a bioremediation substance to enhance the state of the marine environment^39^.

Seaweed farming as a method of cultivation has been practiced for many years but has recently gained popularity in East Asia mainly because it forms a major ingredient in preparing meals like sushi, soups, and salads. This emerging market proves an excellent chance to expand portfolios, as seaweed provides consumers with a naturally brothy, meatless or fishless taste. By 2040, seaweed production could exceed 100 million tonnes of food, while Europe, which currently accounts for only 0.8% of global seaweed harvest, remains in the developing stage of this industry^40^. But, like any major practice of the agricultural industry, then it is imperative that seaweed farming should be made environment-friendly. Experts warn against replicating the environmental mistakes of land farming. Developed guidelines for the sustainable seaweed farming include limited scale and centralized management of farms within communities, use of local seaweed species, and processing of the final products near the farm in an effort to mitigate greenhouse gas emissions. Places like Aird Fada farm which can be found off coast of the Isle of Mull in Scotland advocates for this kind of practices throughout the farming and agriculture in order to avoid any form of interference with the ecosystem. They wish to enhance it; providing further a floating coral to incorporate other flash bio-diverse species, as well as the juvenile queen scallop, crabs, and seabirds^33^. Despite its promise, seaweed farming is not without challenges as rising ocean temperatures, a consequence of climate change, pose a significant threat, with some regions experiencing water temperatures that exceed the tolerance levels of seaweed crops. Additionally, warming waters can promote the growth of pests and diseases that affect seaweed production. For example, biofouling, the accumulation of marine organisms on seaweed lines, has become more prevalent, particularly in warmer waters, reducing crop yields. Farmers are adapting by moving crops to cooler, deeper waters, though this makes them more challenging to manage.

Beyond its role as a food source, seaweed has multiple other applications. It can be used as livestock feed to reduce methane emissions from cattle, as a natural fertilizer to reduce dependence on artificial fertilizers, and even as a biodegradable alternative to plastic. Moreover, seaweed farms contribute to the local economy by creating jobs and supporting coastal communities. Looking ahead, seaweed farming holds the potential to play a key role in addressing global food security and environmental challenges. With careful planning and sustainable practices, seaweed farming could become a cornerstone of a more resilient and eco-friendly food system. By integrating seaweed farming with other agricultural practices, such as filtering agricultural runoff with oysters, the impact of nutrient overload on ecosystems could be mitigated, providing a sustainable cycle that benefits both the environment and local communities.

### Plant-based and lab-cultured meat

**Fig. 9:**
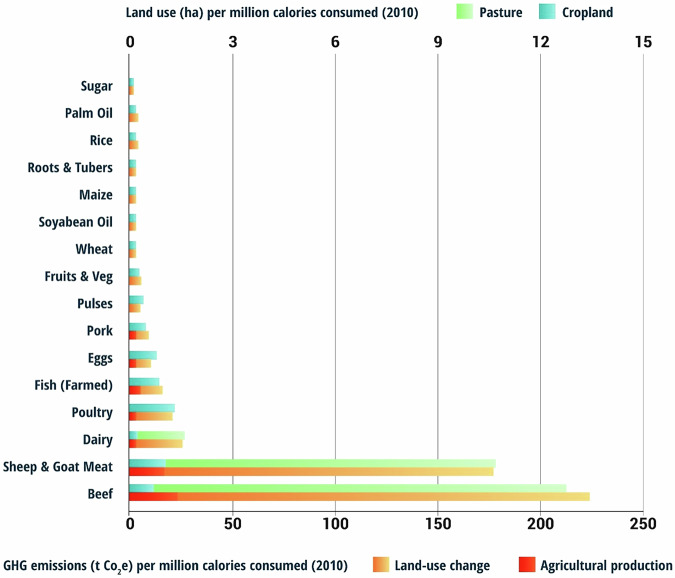
Projected environmental impact of dietary shifts towards plant-based alternatives by 2050^42^.

It is believed that bioinformatics and other technologies can reduce reliance on traditional livestock rearing. It predicted a rise of 88% for the consumption of ruminant meat including beef, lamb and goat by 2050 from the year 2010. Beef is the most popular ruminant meat and globally, it takes 100 times more water and emits five times as much GHG to produce a kilogram of beef than it does to produce plant proteins- beans or peas. If U.S. consumers reduced their daily intake of ruminant meat to just 52 calories—approximately equivalent to 1.5 hamburgers per week—it could result in a 50% reduction in the greenhouse gas (GHG) mitigation gap and nearly eliminate the land deficit, particularly in North America. Achieving this would require halving current beef and lamb consumption levels. Strategies to support this transition include promoting plant-based foods, advancing meat analogs, and implementing policies that encourage plant-based diets, as illustrated in Fig. 9^41^.

Plant protein sources from legumes, grains, nuts, seeds and vegetables can effectively replace animal protein sources, which are resource scarce and out-rightly damaging to the environment. For example, the generation of protein source from plants is often found to consume less water, land and energy than does the generation of protein from meats. Lentils, chickpeas and beans for example are favorite crops because they are proteinaceous and easy to produce in different regions with little inputs. More recently, the so called ‘fake meats’ industry has grown, as consumers have become more conscious of what they are eating and wanting healthier, more sustainable, and, in many cases, cruelty-free products^42^. As a growing number of consumers refrain from eating animal meat due to health, ethical, or environmental concerns, the food service industry has responded by incorporating alternative products into their offerings. The main types of alternatives are plant-based meat and cultured meat, each with unique qualities and advantages. However, this change in diet preferences has actually transformed a whole industry of food production. The concept of plant-based meat is not new, as veggie burgers have been available since the 1980s. However, these early versions were primarily designed for vegetarians and vegans and did not closely resemble burgers made from beef, chicken, or pork in taste or texture. These plant-based products are gradually gaining popularity among non-vegetarians as they closely replicate the taste and texture of real animal meat. Despite the advancements made, it is essential to acknowledge how far these products have evolved over time^43^. Plant proteins undergo thermal and mechanical stress during “high-moisture extrusion,” a process that gives them a meat-like texture. This technique is used to produce plant-based options, such as veggie burgers and meatballs, which restaurants incorporate into recipes or as alternatives to traditional meat dishes. Cultured meat, also known as “in vitro” or lab-grown meat, is derived from tissue culture by growing animal muscle cells and stem cells. It offers the same protein content as beef, pork, or chicken, providing a way to consume real meat without the drawbacks of conventional meat production. However, as it originates from animals, cultured meat may not align with the dietary restrictions of those with health concerns or religious objections to meat. While plant-based meats are increasingly featured on restaurant menus due to the rise of flexitarian diets, lab-grown meat is less common. Its high production cost and limited availability make it an impractical option for most restaurants, and widespread adoption is expected to take time^44^. Lab grown and plant-based meat both have their unique benefits. Lab-grown meat, also known as “cultured meat,” is produced through a different approach. It involves cultivating animal muscle samples and stem cells to grow into a large amount of meat. Unlike plant-based meat, lab-grown meat contains the same nutritional content and animal protein found in conventionally produced beef, pork, or chicken. This offers consumers the opportunity to enjoy real meat without the ethical and environmental concerns associated with traditional meat production. However, since lab-grown meat is derived from animals, it may not be suitable for people with specific health concerns or religious beliefs that avoid meat consumption^44^.

Plant-based meats are increasingly featured on restaurant menus as chefs aim to cater to the growing number of flexitarian diners. In contrast, lab-grown meat is still costly to produce and not yet widely accessible or affordable for most establishments. Once production costs decrease, lab-grown meats could become a viable option for diverse dining experiences. Both plant-based and lab-grown meats offer distinct advantages. Although getting to the mainstream might be slower for cultured meat, products derived from plants are on the rise. At the same time, the inclusion of plant-based meats into iconic meals can simply give restaurants a chance to engage customers with the tastier and healthier picture of a plant-based future of meats. Moreover, the popularity of plant-based meat alternatives, such as those made by companies like Beyond Meat and Impossible Foods, has skyrocketed. These products replicate the taste and texture of traditional meat but are made from plant ingredients like soy, peas, and rice. The production of plant-based meat alternatives produces fewer greenhouse gas emissions and uses significantly less water and land than conventional meat production, making them a more sustainable choice for future food systems.

### Plant-materials-Based 3D printing

Plant-based meat substitutes or replacers are frequently derived from a diverse array of materials, including cereals (e.g., barley and rye), legumes (e.g., mung beans, common beans, and lentils), and oil seeds (e.g., cotton seed and rapeseed)^45^. Nevertheless, the widespread replacement of livestock meat has not yet occurred, despite the development of some innovative plant-based meat analogs. Consumer perception and cost are the primary factors contributing to the absence of replacement^46^. Consequently, numerous recent studies have concentrated on the development of meat analogs using a variety of plant materials. In this regard, researchers have concentrated on pea protein due to its distinctive characteristics, which enable it to provide complementary functions to other constituents^47^. Nevertheless, the gelling capacity of pea protein is reported to be lower than that of soy protein, necessitating the inclusion of salt or the modification of particle size. Nevertheless, the development of meat analogs does not necessitate the use of high-purity soy, which is a common ingredient. This is a result of the presence of numerous constituents that improve its ability to assimilate fat, gel, and retain water. Wheat is another plant material that has garnered attention for its ability to form disulfide protein linkages, which can lead to fibrous proteinaceous structures^48^. This material is frequently employed and has been demonstrated to enhance the viscoelastic and rheological characteristics of analogs^45^. According to Kyriakopoulou et al.^48^ the structure of meat analogs can be enhanced through the application of intense heat or pressure to rapeseed proteins. Lipid ingredients, including vegetable oils (e.g., sunflower oil and canola oil), have also been employed in the formulation of meat analogs to impart flavor and juiciness^47^. Lille et al.^49^ have observed that the aggregation of fibrous networks can obstruct the printer nozzle and result in structures with layer definitions, despite the fact that there are numerous materials derived from plants. Additionally, it may be necessary to incorporate a variety of hydrocolloids into the 3D printing process for meat products. Xanthan gum, alginate, gum arabic, and carrageenan are among the hydrocolloids that are available^50^. Cellulose nanofiber (CNF) and other nano-scaled materials have the potential to be employed in 3DP. The incorporation of CNF in pastes has been demonstrated to enhance the shape stability of 3D structures and reduce the clogging of ends^49^.

The 3D printing of food products with intricate matrices, such as meat, is regarded as a difficult task. The challenge in developing meat substitutes that resemble meat products using only plant materials is even more difficult. Beyond Burger and Impossible Foods, two start-up ventures, have specifically addressed this challenge by developing plant-based meat substitutes. Despite the fact that Impossible Foods (Redwood City, California, US) employs a variety of plant-based constituents, soy leghemoglobin is the primary ingredient used to develop the distinctive meat color, which is due to the pigment protein myoglobin. On the other hand, Beyond Burger (El Segundo, California, US) employs beet juice extract, while another venture has implemented tomato paste^47^. Other natural pigments, including red rice, annatto, paprika, and red peppers, may also be employed^51^. In the realm of 3D printing, NOVAMEAT, a start-up based in Barcelona, Spain, has asserted that it has successfully printed poultry and beef. In 2018, this Barcelona-based company asserted that it had printed sirloin using ingredients such as pea, seaweed, and beetroot juice. Organoleptically, the 3D-printed steak was reported to be comparable to conventional beef steaks. The filaments that were printed had diameters that ranged from 100 µ to 500 µ. The cost-effectiveness of the fabrication was demonstrated by the production of a 50 g steak at a cost of $1.50 USD^52^. Additionally, a company based in Israel has asserted that it has developed plant-based steaks that are composed of plant-based protein, oil, and water. The cost of a tenderloin that was ~200 g in weight was approximately $ 4 USD, as stated in a press release. In addition to beef, they intend to concentrate on other livestock meats, including pork and tuna^52^. Nevertheless, it is essential to acknowledge that the absence of comprehensive public information may result in the company, products, or processes being uncertain about their future. However, the development of 3D meat analogs is impeded by the necessity of texturizing plant proteins, the inadequacy of essential amino acids, and the incorporation of a variety of non-proteinaceous ingredients. Additionally, sausages, patties, and mince comprise the majority of plant-based analogs. It appears that the production of steaks from plant-based materials is a challenging endeavor^53^. Nevertheless, Meat and Livestock Australia introduced the concept of an emulsified red meat ink at a 3D food printing conference in 2017^54^. A snack was recently 3D printed using mushroom, an additional ingredient that bears a resemblance to meat. Due to the aggregation of the intricate network of fibers, the printing of fiber-rich food is regarded as a challenge due to the blockage of printer nozzles. Consequently, mushrooms could be employed as a primary component in the production of meat analogs. Nevertheless, insects serve as sustainable alternatives to plant-derived materials in the development of meat analogs.

### Gen next fish farm

Advancing aquaculture techniques for efficient and sustainable seafood production has become a focal point in an era of rapid technological progress. As global populations surge, ensuring food production meets the growing demand becomes a pressing concern. In this context, aquaculture, the practice of farming marine species, offers a potential solution, continuing a centuries-old relationship between humankind and the sea. For years, we’ve relied on the ocean for nourishment, but overfishing has led to a decline in many marine species. Today, more than half of the world’s wild fisheries are fully exploited, with over 30% at risk of collapse due to overexploitation. The answer may lie not in harvesting from the wild but in responsibly farming ocean life through innovative aquaculture techniques^55^.

It has been noted that aquaculture has become important player in the provision of seafood and is responsible for more than half of the total consumption. Nevertheless, modern trend of fish farming including specifically mariculture incurs a number of environmental impacts. Cages or pens in which closed-cycle fish are in the immediate physical contact with the natural seawater body have been damaging to the environment in many ways. These farms often facilitate the spread of diseases to wild fish populations, and the release of pen-raised fish can disrupt the social structure of natural fish communities. Additionally, the reliance on fishmeal and other feedstuffs for carnivorous farmed fish places significant pressure on global wild fish stocks. With the expansion of aquaculture, closed containment systems—where fish are confined to controlled environments—are increasingly seen as a sustainable solution. Such systems reduce risks such as spread of diseases among the fish, escape of fish and proportional discharge of waste. Nevertheless, there is limited achievement since the systems have several drawbacks for instance high energy costs when maintaining the systems and are also costly when it comes to waste water discharge^56,57^. Since wild fish stocks are already running low, aquaculture supplies must rise by 25% in order to meet the 58% global fish consumption that is expected to occur between 2010 and 2050. Enhancing aquaculture productivity worldwide and tackling today’s environmental issues, such as wetland loss, wild fish replacement in feeds, excessive freshwater use, and water pollution, are necessary to achieve this expansion. These include feed formulation, frequent disease management, water circulatory systems and other pollution-reducing measures, spatial management of new and expanding fish farming units, increased marine fish farming. The implementation of water recirculation systems and other pollution controls, improved spatial planning for new and expanding farms, increasing marine-based fish farming, improving feed quality and disease management, and selective breeding to increase fish growth rates are all important actions^58^.

The development of such closed-contained systems marks a major leap forward in aquaculture. This technology offers a sustainable alternative to traditional fish farming, with the potential to meet the growing demand for seafood without overburdening the ocean. As such systems become more accessible and cost-effective, they could play a pivotal role in reshaping the future of global food production^59^. However, for these advancements to truly revolutionize aquaculture, collaboration between scientists, policymakers, and industries is crucial. Sustainable fish farming can ensure a stable food source for the growing global population, but it will require continued investment, innovation, and consumer demand for eco-friendly practices. The future of fish farming lies not just in the sea but on land, where technology, sustainability, and science converge to create a new era of aquaculture.

### Underutilized crops

The world is confronting a significant challenge: a mounting food crisis exacerbated by a projected global population surge to nearly 10 billion by 2050, placing increased pressure on current agricultural systems. At the same time, agriculture’s influence on biodiversity and land usage exacerbates the situation. Climate change and the loss of natural resources pose unprecedented challenges to the global agro-food value chains. In response to these issues, crop diversification can give a long-term solution for ensuring future food security, encouraging us to look beyond the most well-known crops. Furthermore, dietary tastes and choices have a direct impact on our health, with increased demand for vegetarian and vegan alternatives as plant-based diets gain popularity^60^.

The main issue with current practices in the agricultural sector is that modern agricultural practices predominantly depend on a few energy-dense but nutrient-poor crops, with rice, maize, and wheat accounting for ~60% of the world’s dietary energy supply. This reliance endangers food sovereignty, as genetic erosion driven by climate change and its severe impacts jeopardizes access to staple crops, traditional foods, and income sources for vulnerable populations, including smallholder farmers, children, women, and indigenous communities. On the other hand, underutilized crops offer a solution as they offer even better health benefits, enhanced nutritional value, impressive resistance to the vagaries of climate. We must focus on including the left-out crops into cropping systems of today in order to address problems such malnutrition, scarcity in food, and reliance on singular cropping systems. In reality, a great many of these underutilized plant foods are capable of reducing the over reliance on a few cash crops and become valuable players in food security initiatives in this world^61^. Orphan crops are a way to extend existing crop portfolios, together with sustainable agriculture practices and the promotion of agrobiodiversity can supply a constant food supply that is healthy for human consumption while minimizes environmental change.

Breeding and biotechnological advancements can enhance these crops, promoting agricultural diversification and profitability. Samal et al.^62^ highlighted the potential of underutilized legumes, while Chivenge et al.^63^ explored the nutritional and nutraceutical benefits of rice bean (*Vigna umbellata*). Underutilized tuber crops, due to their high energy content, play a crucial role in ensuring food security, especially in developing countries. Dioscorea, for example, is an important genus that feeds millions of people globally, particularly in tropical and subtropical areas. Chinese yam, which is known for its health advantages, can also be grown in temperate regions, however molecular research on it is still limited. RNA sequencing of two *D. polystachya* tuber variations revealed the involvement of brassinosteroids in tuber formation, providing information that could be used to guide breeding methods^64^. Amaranth and quinoa, both high in critical minerals and gluten-free, are becoming attractive alternatives to traditional cereals. While their genetic diversity is immense, progress toward high-yielding cultivars has been gradual. Another major medicinal plant, Perilla, is widely cultivated in Asia and Li and Siddiqui^64^, examined its nutritional and therapeutic qualities, offering suggestions for further research and development.

Underutilized crops have the potential to address the world’s ongoing food security issues by providing sustainability, nutritional value, and resilience. These crops help to improve food security, protect agrobiodiversity, and build more resilient agricultural systems. While hundreds of indigenous crops are underutilized, embracing them will contribute to a more robust and diverse global food system. These crops are valuable not only for conservation, but also for studying the genetic features that allow them to survive in severe environments. Despite modest development, there is an urgent need to integrate these crops into mainstream agriculture to increase food security and sustainability.

### Hydroponics

Hydroponics has the potential to address a variety of global issues, including climate change, urbanization, and resource depletion. It allows crops to be cultivated under nearly optimal conditions year-round, regardless of external weather or soil quality. This method could deliver fresh, local food to areas with inadequate soil or severe drought, such as Sub-Saharan Africa^65^. Hydroponic farming employs sophisticated climate control systems, including sensors that monitor water, fertilizer levels, and plant transpiration. This precision allows farmers to optimize inputs, resulting in optimum yields and lowest waste. Hydroponic allows crops to be cultivated under nearly optimal conditions year-round, regardless of external weather or soil quality. In addition to being water efficient, hydroponics minimizes the need for pesticides^66^. The controlled environment restricts crop exposure to pests and diseases, decreasing the need for hazardous chemicals that frequently affect ecosystems. Soilless agricultural systems offer a sustainable solution for urban and resource-constrained settings, ensuring cleaner, safer produce with minimal environmental impact. By 2050, food production must increase by 70% to meet the demands of a projected global population of 9.8 billion, 68% of whom will live in cities^67^. Traditional agriculture, which already consumes 70% of freshwater and 38% of non-frozen land, cannot sustainably meet future demands. Expanding farmland by an additional 593 million hectares would threaten critical ecosystems like rainforests, which play a key role in regulating the biosphere and supporting much of the world’s biodiversity. Deforestation driven by agricultural expansion has already led to a 52% decline in vertebrate species and is the second-largest contributor to human carbon emissions.

Hydroponic farming, a type of controlled environment agriculture (CEA), provides a sustainable alternative. It eliminates the need for soil and pesticides, promoting plant growth by directly supplying nutrients, water, and light in a controlled, dirt-free setting. Hydroponics, which uses growing media such as coconut husk and nutrient-infused water, as well as aeroponics (misting plant roots), can be vertical, utilizing 90–99% less land while boosting productivity. Despite its advantages, hydroponic farming poses obstacles, especially for small-scale, beginning producers^68^. Hydroponic systems have high startup costs, which include space, materials, lighting, and climate control technology. Although the initial investment may be substantial, hydroponic farms have the potential to transform unused urban areas into productive agricultural spaces, offering opportunities for local food production and job creation. Consequently, hydroponics could serve as a viable alternative to conventional agriculture. It has the potential to alleviate the future food security dilemma, minimize resource consumption, and mitigate the environmental effect of conventional agricultural operations by leveraging controlled conditions and innovative technologies. However, addressing the financial and logistical hurdles of establishing hydroponic farms will be critical to realize their full potential as a sustainable alternative for the future^69^.

A comparative analysis of the advantages and disadvantages of each alternative food source is presented in Table 4.

**Table 4:** Advantages and disadvantages of alternative food sources

Alternative Food Source	Advantages	Disadvantages	References
Edible Insects	High in protein, low GHG emissions, minimal land use, fast reproduction.	Consumer acceptance issues, regulatory challenges, potential allergenicity.	^28^
Seaweeds	Rich in nutrients, do not require arable land or freshwater, carbon sink potential.	Seasonal availability, heavy metal accumulation, taste and texture barrier.	^193^
Plant-Based Meat	Lower environmental footprint, cholesterol-free, animal welfare-friendly.	Highly processed, taste/textural gaps vs meat, dependency on specific crops like soy/pea.	^48^
Lab-Cultured Meat	Animal-free meat alternative, high protein content, scalable in controlled environments.	High production cost, ethical and regulatory concerns, limited large-scale infrastructure.	^194^
Gen Next Fish Farming	Efficient water use, controlled disease spread, sustainable fish yield.	High energy input, capital-intensive, complex management.	^195^
Underutilized Crops	Climate-resilient, nutritionally dense, support agrobiodiversity.	Market demand low, limited supply chains, lack of awareness.	^196^
Hydroponics	Water-efficient, space-saving, year-round production, pesticide-free.	High setup cost, technical expertise needed, limited crop diversity.	^197^

## Blockchain technology

**Fig. 10:**
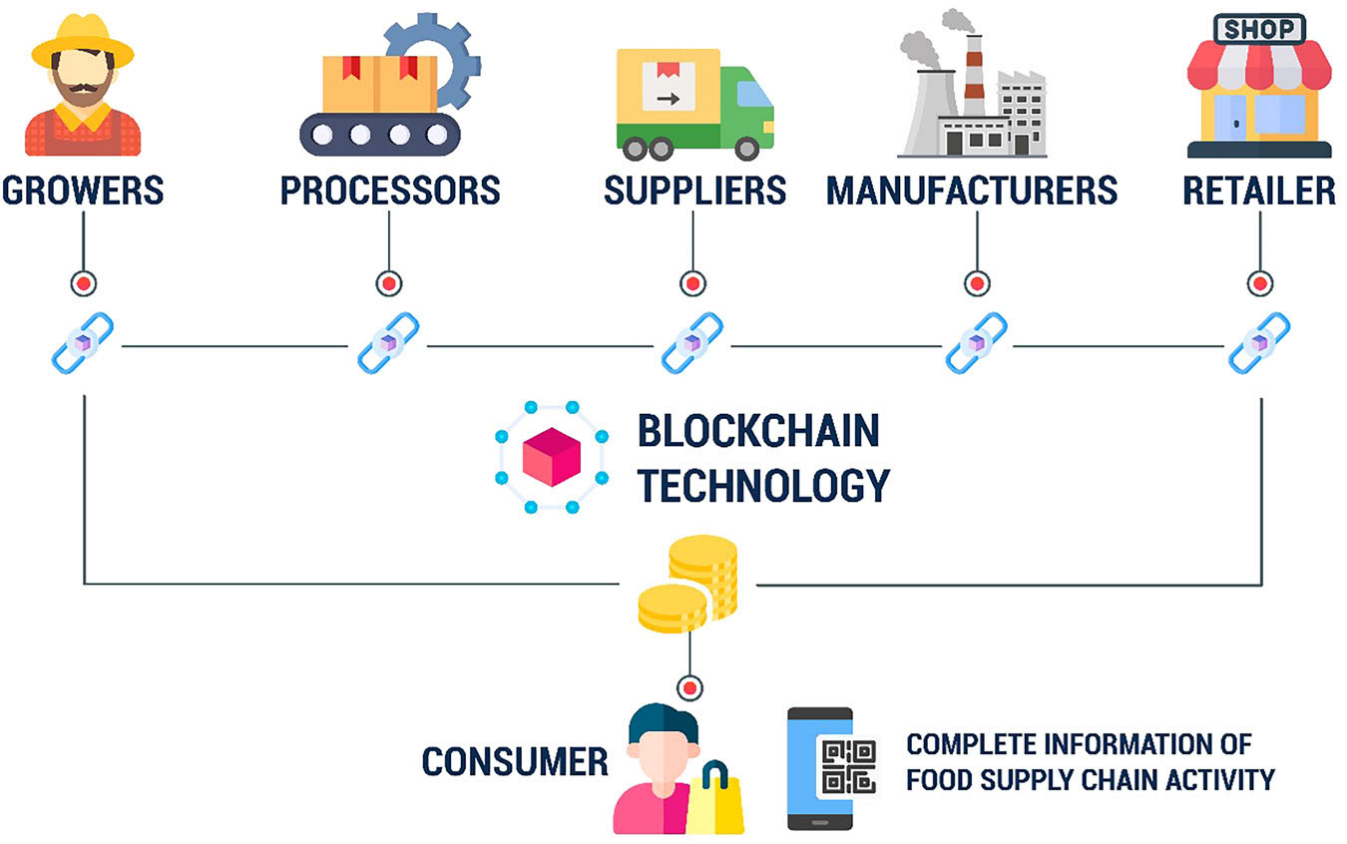
Blockchain technology revolutionizes food safety and sustainability by ensuring transparency, traceability, and efficiency across the entire supply chain, from farm to table^72^.

Blockchain technology is gaining recognition for its ability to alter company operations, notably in the food industry, by improving transparency, traceability, and security^70^. Blockchain is defined as a shared, cryptographically secure, and programmable ledger that no single user has control over, providing a transparent and immutable record of transactions. This technology prevents data manipulation, allowing consumers to trust the origin and movement of food goods across the supply chain. With all stakeholders on the network, including producers, operators, and retailers, blockchain ensures that once a transaction is recorded, it cannot be changed, enhancing food safety and confidence^71^. In the food business, blockchain has the potential of enhancing the supply chain immensely since the profiles of all the members involved together with food safety technologies such as QR codes and labels can be implemented. This makes it easier to detect and eliminate adverse foods that pose danger to the lives of people and thus do not reach the customer’s table^72^. In addition, the issue of food fraud which is ranked at multibillion-dollar globally can be neutralized by blockchain’s clear structure as it maintains records of transactions. It also decreases costs related to inventory tracking and delivery because one no longer has to pay for third-party verifiers as shown in Fig. 10.

Blockchain technology has the potential to completely transform food traceability by guaranteeing the quick and trustworthy interchange of data, giving customers the ability to confirm the provenance of ingredients and evaluate the accuracy of information. When appropriate safety procedures are followed, blockchain facilitates the smooth transmission of data from producers to processors, fostering trust in the veracity of supply chain data. The efficiency of product recall procedures can also be greatly increased by retailers using this technology to detect potentially dangerous products early on^73^. Because blockchain is decentralized, its intrinsic openness guarantees that all parties involved, including producers and processors, have access to constantly updated ledgers. Increased accountability and traceability across the supply chain are fostered by this real-time visibility. As a result, incorporating blockchain technology improves food safety protocols, lowering hazards and guaranteeing a more reliable and safe food system.

Throughout the value chain, blockchain technology improves efficiency, security, and transparency. Better traceability, less fraud, and automated transactions using smart contracts that guarantee equitable payments are all advantageous to producers. Verified product information is made available to customers, guaranteeing safety, ethical origin, and authenticity. Blockchain makes the supply chain more dependable and sustainable for all parties involved by reducing middlemen and boosting trust. By allowing consumers to track food goods, it ensures they are fresh, safe, and sustainably sourced. Walmart used blockchain to track mangoes to their farm in 2.2 s, compared to six days before. Starbucks uses blockchain to track coffee beans, empowering producers and ensuring product authenticity. This technology has great potential despite its drawbacks^74^. However, for blockchain to work effectively, correct data from the source is required. All supply chain parties must agree to use the technology and collaborate in gathering and sharing valuable data. Furthermore, blockchain grants public access to information while ensuring that data ownership remains with the user, who retains control over determining access permissions and the level of detail shared. Therefore, blockchain technology has the potential to transform food safety and sustainability by increasing transparency, traceability, and efficiency throughout the supply chain. However, its success is contingent on the cooperation of all stakeholders and the veracity of the information given.

## Biotechnology in food

Biotechnology is creating waves in the food and farming industries, providing new answers to some of agriculture’s most pressing concerns. As the world’s population rises and environmental concerns become more serious, biotechnology offers new opportunities to improve food supply and make farming more sustainable. This essay will look at the key advantages of biotechnology in these fields and discuss how these breakthroughs are influencing the future of agriculture. Through increased crop yields, improved disease and insect resistance, and the ability to adapt to harsh environmental circumstances, biotechnology has completely changed the food production process. Genetically modified (GM) crops, like pest- and drought-resistant types, boost agricultural productivity overall and lessen reliance on chemical inputs. Furthermore, biotechnological developments such as synthetic biology, microbial fermentation, and tissue culture aid in the creation of sustainable and nutrient-dense food sources. In line with Sustainable Development Goal 2 (SDG-2) to eradicate hunger, these inventions aid in addressing issues related to global food security. Scientists used genetic engineering to create crops that generate more food per plant. For example, genetically modified (GM) crops such as Roundup and Bt cotton Ready soybeans are intended to be more productive. Bt cotton includes a protein that defends it against pests, allowing farmers to increase harvests without using as many pesticides^75^. Research indicates that genetically modified (GM) crops can boost yields by 10% to 20%, a critical factor in addressing the challenge of feeding the world’s growing population. Higher crop yields allow for increased food production while reducing resource inputs, thereby enhancing agricultural productivity and sustainability. Another significant benefit of biotechnology is the development of pest-resistant crops. Unlike conventional agriculture, which relies heavily on pesticides to protect crops, biotechnology provides a safer alternative by engineering crops with natural resistance to pests, thereby minimizing the need for toxic pesticides^76^. For example, scientists developed varieties of rice that are resistant to grain blasts, otherwise the crop could suffer huge losses. These disease-resistant crops not only help maintain food security, but also reduce waste our environment affected by the use of pesticides, making agriculture more sustainable^77^. Scientists can increase the amount of essential vitamins and minerals in crops through a process called biofortification. Vitamin A deficiency is a significant health concern in many developing countries, and Golden Rice presents a potential solution^78^. Modern agriculture emphasizes sustainability, with biotechnology playing a pivotal role in achieving this objective. Innovations in biotechnology are empowering farmers to minimize their environmental impact by reducing the use of resources like water and fertilizers.

These methods not only preserve resources but also improve biodiversity and soil health. By lowering the use of chemicals and promoting a variety of species necessary for preserving an ecosystem in balance, biotechnology promotes soil health. Additionally, it helps farmers make better use of their resources. Precision agriculture is one example of an advanced instrument that uses data to assist farmers in allocating resources in an informed manner. This guarantees more precise use of water, fertilizer, and other inputs, reducing waste and increasing crop yields. While effective resource management raises profitability and fosters long-term sustainability through cautious and responsible stewardship, these innovations help farmers by increasing yields and lowering costs^79^. Biotechnology provides ways to help crops adapt as climate change alters weather patterns and growing conditions. Researchers are creating robust crops that can resist severe weather conditions like floods and droughts. These crops are designed to flourish in harsh conditions and use water as efficiently as possible.

In times of water constraint, for example, crops that can withstand drought assist maintain food production. As we manage upcoming environmental difficulties, such improvements will continue to be crucial for ensuring food security in a changing climate. Biotechnology has many advantages for food and agriculture, such as higher crop yields, better nutritional value, improved resistance to pests, and the encouragement of sustainable practices. These developments are changing agriculture and helping us deal with the problems brought on by population growth and environmental change. Increasingly, biotechnology will be essential as we work to generate enough food while preserving the environment. To ensure a productive and sustainable agricultural future, these technologies must be continuously researched and adopted.

## Robotics in food

The application of robotic systems to a range of food industry operations, including processing, packaging, serving, and preparation, is known as food robotics (Table 5). These robots are made to automate monotonous jobs, increase productivity, lower operating expenses, and handle food with more accuracy and hygienic conditions. The market for food robotics is growing as businesses look for creative ways to meet strict safety standards, solve labor shortages, and expedite production. The global food robotics market is anticipated to develop at a compound annual growth rate (CAGR) of 11.5% from 2021 to 2033, from USD 3.0 billion in 2023 to USD 8.9 billion by 2033, according to a recent analysis^80^. The food robotics market is expanding significantly due to a number of important considerations. The main factors driving expansion are the need for more effective production lines, the growing demand for packaged foods, and the rising standards for food safety^81^. Further driving market expansion are technological developments in robotic systems, such as improved sensors, machine learning, and artificial intelligence, which make it possible to automate increasingly complicated jobs. The expansion in food production worldwide and the requirement for automation to uphold constant safety and quality requirements are driving up demand for food robotics. Robotic systems are being used more frequently by industries to address labor shortage issues and reduce human interaction with food during processing and packing. This is especially important in the wake of health emergencies like the COVID-19 pandemic^82^.

**Table 5:** Application of robotics in food industry^198–200^

Category	Application	Examples of Robotics systems	Key Benefits
Food Processing	Cutting, slicing, and dicing of fruits, vegetables, meat, and bakery products	ABB Flex Picker, Fanuc robots	Precision, consistency, reduced waste
	Automated mixing and kneading in baking and dairy industries	Robotic dough kneaders, dairy mixing arms	Uniform product quality, labor cost reduction
	Deboning and filleting in meat and seafood processing	Intelligent robotic butchers	Intelligent robotic butchers
Food Packaging	Automated sorting, grading, and inspection of food products	Vision-guided robots, AI-based robotic sorters	Improved quality control, reduced errors
	Pick-and-place systems for food items into containers	Delta robots, SCARA robots	Faster operations, increased efficiency
	Robotic palletizing and depalletizing for bulk packaging	Robotic arms with AI integration	Space optimization, reduced manual labor
Food Serving	Robot-assisted food delivery in restaurants and cafes	Self-driving robots (Kiwibot, Starship)	Contactless service, faster delivery
	Automated cooking and grilling	Flippy (robotic burger flipper)	Reduced operational costs, enhanced consistency
	Self-service robotic kiosks for coffee, ice cream, and snacks	Briggo coffee robot, Sally salad-making robot	Customization, improved efficiency
Food Preparation	Automated dough rolling, pasta making, and sushi rolling	Sushi-making robots, dough sheeters	Uniform product size, time savings
	Smart food dispensing for beverages and sauces	Sauce dispensing robots, coffee-making robots	Precision, portion control
	AI-powered food assembly (e.g., burger, pizza making)	Pazzi pizza robot, Creator burger robot	Speed, consistency, cost savings
Quality control & safety	AI-driven robotic inspection for contamination and spoilage	X-ray and vision-based robots	Enhanced food safety, early defect detection

**Fig. 11:**
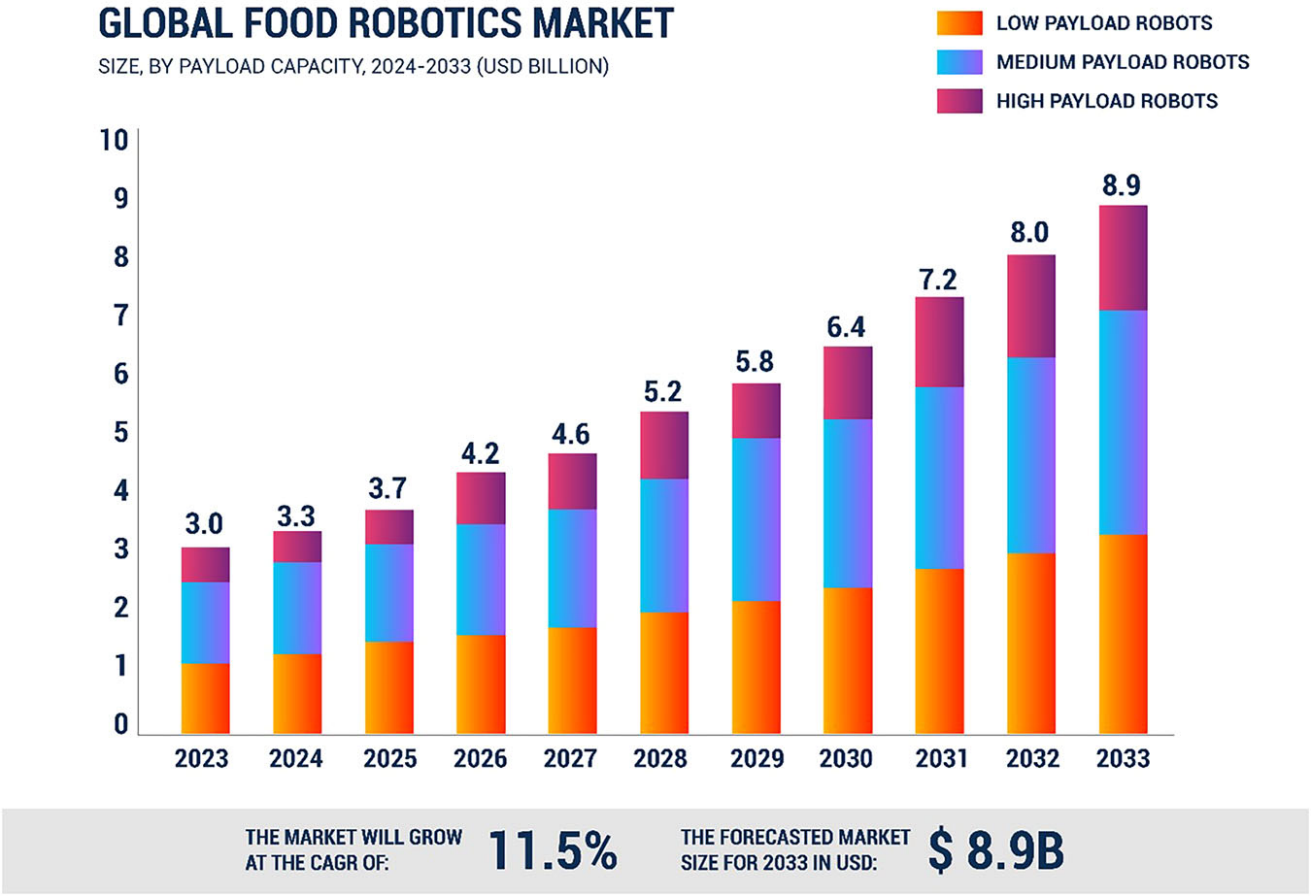
Key Agricultural Parameters Tracked Using Artificial Intelligence^203^.

As shown in Fig. 11 Food Robotics Market is positioned for substantial expansion, exhibiting a projected compound annual growth rate (CAGR) of 11.5% from 2024 to 2033. It is anticipated to escalate from USD 3.0 billion in 2023 to a remarkable USD 8.9 billion by 2033. Asia-Pacific, which led the market in 2023, accounted for 42.7% of the share and generated USD 1.8 billion in revenue. This dominance can be attributed to rapid industrialization, increased investments in automation technologies and a burgeoning food processing sector, particularly in countries such as China, Japan and South Korea. The Articulated Robots sector captured 34.9% of the market share in 2023, however, Medium Payload Robots dominated the payload category with 49.1%. Palletizing emerged as the most significant application, representing 25.5% of the market share^83^. Beverage companies contributed nearly 23% of the total market value, which is noteworthy. Automation trends indicate that 88% of businesses intend to adopt robotics to improve efficiency and approximately 3 million robots are currently operational worldwide, with over 400,000 new units added each year. The industrial robotics market alone is estimated to yield USD 43.8 billion annually^84^.

In the Asia-Pacific region, automation adoption is further encouraged by governmental initiatives, such as China’s “Made in China 2025” strategy, which promotes robotics in food production. North America and Europe, however, also exhibit strong market activity—driven by the demand for packaged foods and stringent food safety regulations, respectively. Although there are differences among these regions, the underlying trends remain consistent because they all seek to enhance efficiency in food production. This convergence highlights a global shift towards automation, but the pace of adoption varies significantly across different areas. AI integration in food robotics may increase performance, quality, and waste and can be used for resource management, food contamination prevention, predictive maintenance, supply chain management, etc. AI technology is expected to alter the food sector by adding distinctiveness and sustainability to products. In 2023, Yaskawa Electric acquired Doolim-Yaskawa to improve food robotics. OMRON Corporation and IBM Japan’s joint venture is expected to revolutionize production with data. The advancements in Michigan will enable ABB to address the increasing demand for robotics in the food and beverage industry. Other notable trends in food robotics include Autonomous Mobile Robots (AMRs), Robotics-as-a-Service (RaaS), and Artificial Intelligence (AI) as they improve food industry, stock supply, and sanitation performance. Reusing or refurbishing industrial robots contributes to the circular economy. Food robotics is a rapidly growing field that promotes efficiency, food safety, and flexibility in the food business^85^. The increase in labor costs and shortage in many industrialized nations that compels the food processing sectors to adopt automation is the foods robots growth factors. This review article also points out that the pursuit of automation also plays a huge role in the adoption of robotics in the food processing industry in terms of both costs and productivity. This growth is also driven by the increasing demand for food to be delivered more quickly, safely, and reliably^81^. The market for food robotics offers a wide range of prospects, from the creation of robots that can safely handle fragile food items to advancements in robotic sorting and packaging systems. In emerging areas, where automation in food processing is still in its infancy, there is certainly room for expansion. Businesses in the food sector have a lot of chances to innovate and increase their operational efficiency as robotic technologies develop and are incorporated into increasingly complicated food preparation operations.

## Artificial Intelligence (AI) in agriculture

One of the main areas of computer science research is artificial intelligence (AI). AI’s broad range of applications and quick technological advancements are making it widely used. This is due to its strong application in solving problems, especially those that humans and conventional computing structures are unable to handle effectively. Approximately 30.7% of the world’s population works directly in agriculture, which occupies 2781 million hectares of land. From seeding to harvesting, such an enterprise has numerous obstacles, making it difficult to function smoothly. Infestation by pests and diseases, poor chemical application, poor irrigation and drainage, weed control, yield forecast, etc. are the main problems^86^.

In 1983, the use of computers in agriculture was first documented^87^. From databases^88^ to decision support systems^89^, various strategies have been proposed to address the current issues in agriculture. When it comes to accuracy and robustness, AI-based systems have been determined to perform the best among these alternatives. Situations in agriculture are dynamic and cannot be generalized to imply a shared answer. AI techniques have enabled us to capture the intricate details of each situation and provide a solution that is best fit for that particular problem. Various AI approaches are being developed to solve increasingly complicated challenges.

### Agriculture parameters monitored by Artificial intelligence (AI)

**Fig. 12:**
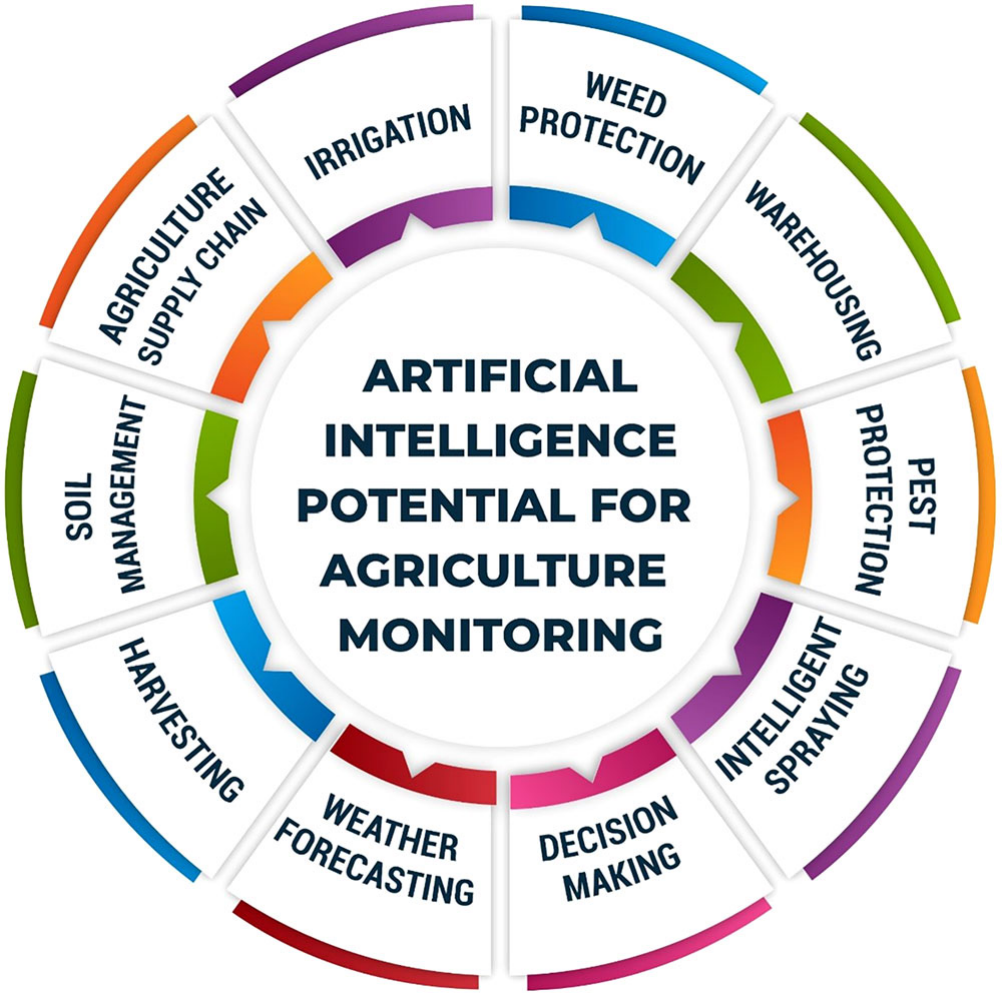
Food Robotics markets growth trajectory year over year^85^.

Agriculture is a labor-intensive industry, which is why it is unsurprising that there is a labor shortage. Nevertheless, automation contains the potential to resolve this issue. These include AI-based harvesting robots, clever irrigation, spraying, and fertilizing systems, as well as auto-driver tractors. For software companies, it may be challenging to ensure that producers comprehend the entirety of the AI system. In agriculture, AI is employed for the purpose of deficiency detection, pest management, health monitoring, and field harvesting. In the agricultural sector, ML (Machine learning) and AI (Artificial intelligence) are being used to replace antiquated forecasting and intelligence methodologies^90^. AI enhances the farms’ adaptability by introducing state-of-the-art technologies to the field. Biosensors have also enabled the monitoring of soil fertility and moisture. However, raw data is collected and a variety of methodologies are employed in place of fundamental linear regression models. The computation and prediction of past weather trends with non-linear dependencies are possible with neural networks. As a consequence, AI can be employed to sow the seeds at the appropriate time for essential commodities such as rice, wheat, and maise, which are predominantly grown and require heavy rainfall^91^. In Fig. 12, certain agricultural parameters that are monitored by AI are shown.

Farms generate thousands of data points each day regarding temperature, soil, water usage, weather, and other variables. AI and ML models utilize this data in real-time to acquire insightful knowledge, including the optimal planting time, the selection of commodities, and the selection of hybrid seeds for increased yields. Precision agriculture is facilitated by AI systems, which improve the overall quality and accuracy of the harvest. The early detection of pests, diseases, and nutritional deficiencies in agriculture is facilitated by AI technology^92^. AI sensors can identify and target vegetation prior to the selection of the appropriate herbicide for the region. As an outcome, the utilization of herbicides is diminished, resulting in cost savings. Numerous technology companies have developed machines that employ artificial intelligence (AI) and computer vision to precisely monitor and spray weeds. In disciplines as diverse as architecture, finance, medicine, sales, and marketing, AI has made significant advancements. In the present day, crops are irrigated, secured, and fertilized using devices that are based on advanced technologies, including AI, ML, and the IoT (Internet of things)^93^.

In order to achieve the intended yield, crops must be watered at the appropriate time. The process of watering on a large scale by a small number of producers became intricate due to a lack of resources. Intelligent irrigation systems have been implemented. These systems are primarily composed of two components: a hardware implementation that utilizes IoT devices and an ML model to determine the soil’s moisture content. The use of the information informs ML’s precision technology, drones, satellite imaging, remote sensing, and crop and soil monitoring systems. Robots with autonomy are being developed to perform traditionally labor-intensive tasks, such as harvesting crops, more efficiently and effectively than traditional human laborers. Nano-based sensing mechanisms and smart sensors are currently being developed and tested to facilitate the early and precise detection of infections, allergies, chemicals, and pollutants in foods, plant and animal production systems, water, and soil^94^.

AI has facilitated the accumulation and analysis of millions of data points generated by agricultural land on a daily basis. With the assistance of AI, farmers can now make informed decisions in real time, thereby resolving an age-old issue. Farmers have the ability to anticipate weather conditions in advance, which enables them to select the optimal time to fertilize their fields. AI has also resolved the issue of water consumption. AI has enabled the assessment of hybrid seeds and their yields prior to their application in the field, thereby increasing productivity and minimizing the likelihood of failed harvests. The majority of farms are confronted with a skilled labor scarcity as a result of the limited number of individuals who pursue a career in agriculture. Initially, farms necessitated a significant number of workers, the majority of whom labored seasonally to harvest crops. Nevertheless, there was a labor deficit as humans transitioned from an agrarian to an urban and suburban society. The shortage was mitigated by AI agents^95^.

They are emerging agricultural technologies that facilitate the collection of data that producers can use to optimize and monitor their crops. Additionally, it is perpetually updated to reflect the ever-changing environmental and ecosystem factors. Technological advancements are indispensable to the contemporary agricultural sector, as evidenced by the emergence of new agricultural technologies. The implementation of novel agricultural technologies facilitates the attainment of agricultural sustainability objectives. AI technology is aiding a variety of industries in enhancing productivity and efficiency. AI is aiding producers in the reduction of adverse environmental effects and the enhancement of efficiency in agriculture. Therefore, the agriculture industry actively embraced AI in order to alter the overall outcome. This technology is applied extensively to optimize production and operational processes in the agricultural, culinary, and bio-system engineering sectors, as well as to address a variety of farming industry challenges^96^.

Crop cultivation can be facilitated by the collection of data from sensors, drones, and satellites. The information collected can be analyzed using AI in farming, which enables producers to make more informed decisions. The analysis of a plant’s biomass and characteristics can be facilitated by the use of AI in phenotyping. AI technology can be enhanced to identify the specific causes of these diseases by analyzing changes in plant biomass and external factors to identify similar patterns between diseased crops^97^. Farmers can enhance their overall yield by identifying parasites and impurities in their crops. The hydrological, climatological, and agronomic equilibrium are all influenced by the water management practices of agriculture. The most sophisticated ML-based applications to date are associated with the estimation of evapotranspiration on a daily, weekly, or monthly basis. This enables the more efficient use of irrigation systems, while the daily dew point temperature prediction assists in the identification of anticipated weather phenomena and the estimation of evapotranspiration and evaporation^98^.

The speed of disruption will be accelerated by the immense potential of AI, which will substantially alter the process of transporting food from the paddock to the plate. Agriculture is currently experiencing a digital revolution. The potential of new agricultural regions will be unlocked by AI, and these new firms will require employees. AI is making the controlled farming of established items such as leafy greens and novel products like insects more practical and affordable^99^. This technology has the potential to help producers address a variety of challenges, including climate change, water availability, and pests. AI employs ML, Deep Learning, and other processes to generate useful predictions that can assist producers in making informed crop decisions, due to the vast amount of data that is made available. AI is capable of analyzing vast quantities of data with unparalleled precision, a feat that is not possible for humans. Numerous farmers are employing AI-powered robots to execute duties that were previously dependent on human labor. The majority of individuals are refraining from engaging in agricultural labor as a profession as a result of the growing urbanization. This has resulted in a decrease in the availability of human labor for agricultural duties^100^.

AI has the potential to facilitate a plethora of technological advancements in the agricultural sector. This includes the utilization of cameras and other sensors, the internet of things, data analytics, and consulting services, among other things. AI in agriculture will become proficient enough to produce more precise predictive insights by analyzing a diverse array of data sources, such as weather, soil, crop performance, and temperature^98^. By swiftly identifying plant maladies and efficiently administering agrochemicals, AI has the potential to improve crop management and yield in agriculture. Machine learning (ML) has the potential to facilitate the rapid phenotyping of plants, the assessment of soil composition, the forecasting of yields, the prediction of weather, and the monitoring of agricultural activities. An increasing number of farmers are incorporating AI, IoT, and other technological advancements to improve the productivity of their land^97^.

AI is assisting farmers in the automation of their agricultural operations and is also progressing toward precision cultivation, which aims to enhance the quality and quantity of crops while using fewer resources. Businesses that are interested in improving AI-based products or services, such as drones, automated machine manufacturing, and agricultural training data, will benefit from future technological advancements. This will enable the world to confront the challenges associated with the food supply for an expanding population. The future of AI in agriculture will necessitate a substantial emphasis on universal access, as the majority of cutting-edge technology is exclusively employed on large, well-connective farms. The future of ML-automated agricultural products and data science in farming will be ensured by expanding the reach and connection to small farms in remote regions worldwide. AI will be beneficial and effective in the agriculture industry due to its ability to optimize resource utilization and efficiency, as well as to address the resource and labor shortage to a significant extent. This technology will also be instrumental in the research and development of horticulture.

## Impact of climate change, dietary shifts, and food production on future sustainable food systems

Global food security is greatly impacted by climate change since it modifies precipitation levels, temperature patterns, and the frequency of extreme weather events. Because of these changes, it is crucial to create resilient agricultural systems that impact crop output, soil health, and water availability. Staple crops like wheat, rice, and maize might yield less when temperatures rise, and crop failures are more likely when weather patterns are unpredictable. Adoption of sustainable food production techniques including climate-smart farming, agroecology, and precision agriculture is necessary to lessen these effects. Food security in the face of climate change can be ensured by strategies including soil carbon sequestration, controlled-environment agriculture, and genetic adaptations for climate resilience^101^.

Future food systems are also being significantly shaped by changes in consumption trends and dietary patterns. Environmental sustainability may be aided by the increasing popularity of plant-based diets, substitute protein sources such as mycoproteins, insect-based proteins, and lab-grown meat, as well as a decreased dependency on intensive animal husbandry. Conventional cattle farming contributes significantly to deforestation, excessive water use, and greenhouse gas emissions. By reducing the prevalence of diet-related diseases including obesity, diabetes, and cardiovascular ailments, adopting more sustainable diets can improve public health outcomes, reduce the carbon footprint of food production, and consume less land and water^102^.

Furthermore, in order to fulfill future demands while maintaining sustainability, food production itself needs to change significantly. Long-term agricultural output can be greatly increased by implementing regenerative agriculture, which aims to boost biodiversity, restore soil health, and utilize fewer chemical inputs. A more effective and sustainable food chain can result from circular food systems that reduce waste through food recycling, composting, and upcycling of byproducts. In urban areas, innovations like hydroponics, vertical farming, and bioreactor-based food production can increase food supply while utilizing less space. Global food sustainability can also be enhanced by lowering food loss and waste at different points in the supply chain, such as harvesting, processing, distribution, and consumption^103^.

Building a resilient food system requires an all-encompassing strategy that incorporates biotechnology, climate adaptation tactics, and dietary changes. To put these reforms into effect and advance sustainable food practices, stakeholders in the food sector, researchers, and policymakers must work together. The key to guaranteeing a safe and sustainable food supply for future generations will be to invest in research, education, and technology-driven solutions.

## Ensuring food security and sustainability: innovation preservation technologies for extending food longevity

**Fig. 13:**
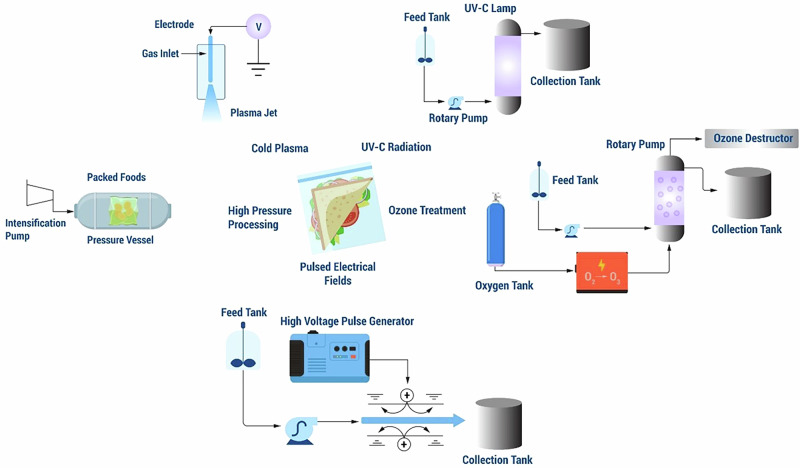
Illustrative overview of novel food preservation technologies, showcasing methods such as cold plasma, UVC radiation, ozone treatment, high-pressure processing, and pulsed electric fields, along with their mechanisms and equipment setups used to inactivate microorganisms and enhance the shelf life of food products^204^.

Innovative preservation technologies are crucial for maintaining the quality, safety, and nutritional value of food products, as well as for extending their shelf life. These technologies encompass a range of methods, including physical, electromagnetic, and biological techniques, and are applied across various stages of the food supply chain. This synthesis highlights the key insights from recent research on innovative preservation technologies. Figure 13 provides an overview of the different innovative preservation technologies for extending the shelf-life of food.

### Cold plasma

Cold plasma technology involves the use of ionized gas at room temperature to inactivate microorganisms on food surfaces. Plasma is produced by imparting energy to a gas, which induces the ionization of gas molecules and the formation of reactive species such as ions, electrons, radicals, and UV Photons^104^. These reactive species cause cellular damage and mortality by interacting with microbial cell membranes, DNA, and proteins. Cold plasma is particularly well-suited for the preservation of heat-sensitive foods due to its non-thermal nature, which effectively inactivates microorganisms without infringing on the food’s nutritional and sensory properties^105^.

Various food products have been successfully treated with cold plasma to assure their safety and extend their shelf life. As an illustration, it has substantially diminished the microbial burden on fresh produce, including strawberries, tomatoes, and leafy greens, thereby extending their shelf life without altering their texture, color, or flavor^106^. Cold plasma was employed on a variety of fruit fluids, including apple^107^, pomegranate^108^, and others^109^. In order to enhance the safety of meat and poultry products while preserving their sensory properties, cold plasma has been employed to decontaminate and reduce the presence of pathogens such as Salmonella and E. coli (Xiang et al.). Furthermore, the application of cold plasma to dairy products, such as milk and cheese, has been used to eliminate spoilage microorganisms and pathogens, thereby enhancing safety and extending the expiration life^110^.

One of the primary benefits of cold plasma technology is that it does not rely on chemical preservatives, thereby reducing the risk of chemical residues on food products. In this way, it becomes a preservation method that is more consumer-friendly and natural. Cold plasma is a non-thermal process that can be applied to heat-sensitive foods without affecting their nutritional and sensory properties. Nevertheless, some research has discovered that prolonged exposure can result in modifications to certain sensorial aspects, such as color^111^ and composition, specifically the fatty acid profile^112^. Additionally, cold plasma is highly effective in the inactivation of a diverse array of microorganisms, such as fungi, viruses, and bacteria, thereby improving the safety and shelf life of food^113^.

In summary, cold plasma technology provides a promising alternative to conventional food preservation methods by combining effective microbial inactivation with minimal impact on food quality. Nevertheless, the pervasive adoption of this technology in the food industry will be contingent upon the resolution of the challenges of equipment cost, scalability, and regulatory approval.

### Pulsed light technology

Pulsed light technology (PL) preserves food by using intense, brief pulses of broad-spectrum light, predominantly in the ultraviolet (UV) range^114^. This high-energy light swiftly inactivates microorganisms on the surface of food products by damaging their DNA and cellular structures, resulting in microbial death^115^. The treatment is both effective and rapid, as the pulses are typically delivered in microseconds, without substantially increasing the temperature of the food^116^. This non-thermal process is particularly beneficial for the preservation of the nutritional and sensory properties of heat-sensitive foods.

The versatility and potential of pulsed light technology have been effectively demonstrated through its application to a diverse array of food products. The presence of pathogens such as *E. coli* and listeria on the surface of fruits and vegetables can be reduced by pulsed light treatment in raw produce^117^. In the dairy industry, pulsed light has been employed to reduce microbial contamination on the surface of cheese, thereby extending its shelf life and improving its safety without compromising its sensory properties^118^. The technology has also been implemented in the production of livestock and poultry, where it has been successful in minimizing surface microbial contamination, thereby enhancing the overall safety and shelf life of these products^119^.

Despite these advantages, several challenges are associated with using pulsed light technology. A potential disadvantage is the restricted effectiveness of light on the surface of food products due to its limited penetration depth^120^. Due to this, it may not be appropriate for foods with intricate surfaces or internal contamination^121^. In addition, the efficacy of pulsed light can be impacted by the physical characteristics of the food, including color, surface texture, and moisture content. Consequently, it may be necessary to adjust the treatment parameters for various products. For instance, foods with larger surface areas, such as minced garlic, exhibit a higher degree of microbial inactivation than those with smaller or rougher surfaces, such as peeled garlic and Manila clams^122^. Nevertheless, Manila clams’ rough surfaces exhibit a lower level of microbial inactivation than finer surfaces, such as those of squid^123^. Furthermore, the low water activity and opaqueness of opaque and irregular surfaces, such as those of seeds and pulverized foods, present obstacles to PL treatment. Nevertheless, optimized PL conditions can still result in substantial microbial reductions^122^. Another obstacle is the equipment’s initial cost, which may be prohibitively expensive for lesser food producers. Lastly, regulatory obstacles and the necessity of thorough safety assessments may arise to guarantee that pulsed light-treated foods satisfy all health and safety regulations^124^.

### High-Pressure Processing (HPP)

High-pressure processing (HPP) is a non-thermal food preservation technology that employs extremely high pressure, typically between 300 and 600 MPa, to inactivate microorganisms and enzymes in food. This process works by subjecting food products to high pressure in a water-based medium, which disrupts the cellular functions of microorganisms without significantly increasing the temperature. As a result, HPP effectively inactivates pathogens and spoilage organisms while preserving the food’s nutritional and sensory qualities^125^. The mechanism of HPP relies on the principle that high pressure disrupts the integrity of microbial cell membranes, leading to cell lysis and death^126^. Additionally, high pressure can denature enzymes that cause spoilage, thereby extending the shelf life of food products^127^. The process is uniform and instantaneous, ensuring that all parts of the food product are subjected to the same pressure, which enhances its effectiveness. HPP stands out as a compelling alternative to traditional thermal processing methods, addressing the increasing consumer demand for minimally processed foods that retain their natural flavors, textures, and nutritional value^128^.

HPP significantly extends the shelf life of various food products by reducing microbial loads and preventing spoilage on meats^129^, fish^130^, beverages^131^, fruits^132^, and dairy^133^. The reduction in spoilage microorganisms results in prolonged freshness and shelf life, which are crucial for both retailers and consumers. The extended shelf life also contributes to reducing food waste, aligning with sustainability goals and providing economic benefits to food producers.

Combining HPP with other preservation methods, such as natural antimicrobials or essential oils, can enhance microbial inactivation, allowing for milder HPP conditions and reducing processing costs^134^. This synergistic approach leverages the strengths of different preservation techniques, offering a more effective and economically viable solution for food safety and quality. For instance, the combination of HPP with natural antimicrobials can result in more efficient microbial control, enabling the use of lower pressures or shorter processing times.

Despite its advantages, HPP is not universally applicable to all food types. Certain dairy and animal products, as well as shelf-stable low-acid foods, may not be suitable for HPP treatment^135^. Additionally, further research is needed to fully understand the scientific theories behind HPP and to optimize processing parameters for different food products^136^. The limitations in applying HPP to a broader range of food products highlight the need for ongoing research and innovation in this field.

### Ultraviolet (UV) radiation

Ultraviolet (UV) radiation, specifically UV-C light, is a non-thermal food preservation technology that inactivates microorganisms on the surface of food products^137^. Singh et al.^138^ have demonstrated that UV-C radiation, which has wavelengths ranging from 200 to 280 nm, is highly effective in entering microbial cells and damaging their DNA, thereby preventing replication and inducing cell death. This approach is effective against a wide range of microorganisms, such as fungi, viruses, and bacteria. UV treatment is appropriate for fresh and minimally processed foods due to its non-thermal nature, which guarantees the preservation of the food’s nutritional and sensory qualities^139^.

UV radiation is employed in a diverse array of food preservation applications. It is frequently employed to sterilize fresh produce, juices, and water^140^. For example, UV treatment reduces the microbial burden on the surface of fruits like apples and tomatoes, thereby preserving their quality and extending their shelf life. UV radiation effectively diminishes microbial contamination in liquid products such as milk and apple cider, thereby improving their safety and shelf life^137^. In the processing of meat and poultry, UV treatment is also employed to reduce surface microorganisms without compromising the sensory properties of the products^141^. Furthermore, the food packaging industry employs UV radiation to sterilize packaging materials, thereby preventing the introduction of contaminants into the food products^141^.

The primary benefit of UV radiation is its ability to inactivate microbial organisms without the use of chemicals, which is consistent with the growing demand for minimally processed and natural foods among consumers. UV treatment is a cost-effective solution for numerous food producers due to its ease of integration into existing production lines and relatively low operational cost.

The composition of foods can be substantially altered when UV treatment is applied incorrectly. Protein digestion issues, antioxidant damage, lipid oxidation, and changes in color and flavor are among the potential modifications^142^. UV radiation has the potential to degrade essential nutrients, which extends its influence on food quality. For example, UV exposure can result in substantial losses of photosensitive vitamins, including vitamin C, B12, B6, B2, folic acid, and fat-soluble vitamins A, K, and E2^143^. The nutritional value of the food is not the only factor that is impacted by the degradation of these vitamins; it can also affect the overall sensory attributes of the food, resulting in less desirable products for consumers. Therefore, although UV-C light can be a beneficial instrument for food preservation, its application must be meticulously monitored to prevent any negative impacts on the quality of the food. However, UV radiation continues to be a promising and adaptable technology for the purpose of extending the expiration life of food and improving its safety, despite these obstacles.

### Ozone treatment

Ozone treatment is an environmentally favorable and potent technique employed in the food processing industry to kill microorganisms, including bacteria, viruses, and fungi^144^. Khadre et al.^145^ have demonstrated that this method utilizes the potent oxidative properties of ozone (O_3_) to disrupt the cellular components of pathogens, thereby extending the shelf life and assuring food safety without the presence of undesirable residues.

The reaction between intracellular enzymes and cell membranes of ozone is highly effective in the inactivation of a wide range of microorganisms, such as bacteria, fungi, and viruses. The microorganisms are effectively neutralized by ozone’s capacity to oxidize essential cellular components, which accounts for its high efficacy^146^. Ozone is a safe alternative for food processing due to its decomposition into oxygen, which leaves no hazardous residues^147^. This is one of its significant advantages. This aspect of ozone treatment is designed to address the concerns of consumers and regulators regarding the presence of chemical residues in food products.

In order to regulate microbial growth and prolong the shelf life of a variety of food products, such as fruits^148^, vegetables^149^, dairy products^150^, and cereals^151^, ozone treatment has been applied successfully. Ozone treatment has been shown to significantly reduce microbial counts in dried figs^152^, red bell peppers^153^, strawberries^154^, and watercress^155^. These applications demonstrate the versatility and efficacy of ozone in a variety of food types, rendering it a valuable instrument for a variety of sectors of the food industry.

When the appropriate dose and duration are established, ozone treatment will typically preserve the sensory and nutritional integrity of food products. Nevertheless, unwanted modifications in food quality may result from improper use or high concentrations. Ozone is an eco-friendly and cost-effective substitute for conventional chemical sanitizers and pesticides, which mitigates environmental concerns and chemical residues^146^. Its application in food processing not only improves food safety and extends shelf life, but also contributes to more sustainable food production practices by reducing dependence on synthetic compounds.

### Pulsed electric Fields (PEFs)

Pulsed electric field (PEF) technology entails the application of high-voltage electric fields to food products in small amounts, with a typical range of 20 to 80 kV/cm^156^. Electroporation is the process by which these electric fields generate cavities in the cell membranes of microorganisms^157^. The loss of cell viability and subsequent microbial inactivation are the results of the disruption of cell membrane^158^. PEF is a non-thermal method, which means that it does not substantially increase the temperature of the food during processing^159^. This property is especially advantageous for the preservation of the sensory and nutritional properties of food, as it averts the degradation of heat-sensitive compounds, including vitamins and flavor components.

PEF technology is primarily employed for liquid and semi-liquid edibles because it is simple to apply uniform electric fields. Milk^160^, fruit fluids^161^, and liquid egg products^162^ have all been preserved using this method. For example, PEFs have been demonstrated to effectively extend the shelf life of orange juice^163^ and apple juice^164^ while preserving their fresh flavor, color, and nutritional content, which can be degraded by conventional thermal pasteurization methods. PEFs have been employed in the dairy industry to process milk, which has led to a substantial decrease in microbial burden without disrupting its flavor, texture, or nutritional properties^165^. PEF treatment has also been advantageous for liquid egg products, which are particularly susceptible to microbial contamination, as it has led to longer-lasting and safer products^166^.

As a food preservation method, PEFs provide numerous substantial benefits. One of the primary advantages is their capacity to inactivate microorganisms without applying substantial heat, thereby preserving the quality of heat-sensitive foods. This non-thermal feature guarantees that the sensory and nutritional properties of food are preserved, rendering PEF-treated products more appealing to consumers who prefer foods that are nutritionally intact and have a fresh taste. In addition, PEFs are energy-efficient, necessitating less energy than conventional thermal pasteurization procedures. This can result in potential cost savings and a diminished environmental impact^167^. Furthermore, the continuous application of PEFs during the pumping of liquid fluids improves the efficiency of food processing operations due to their relatively brief processing time, which can be completed in seconds^168^.

Nevertheless, the implementation of PEF technology is accompanied by a number of obstacles. The high cost of the apparatus, which includes the pulse generators and treatment chambers, can serve as a deterrent to adoption, particularly for small and medium-sized food producers^169^.

### Smart packaging

Smart packaging technologies, such as freshness sensors and time-temperature indicators, are also acquiring popularity. Real-time monitoring of food quality is facilitated by these innovations, which contribute to the reduction of waste and the preservation of food safety. Intelligent packaging systems that monitor environmental conditions and food quality can alert consumers when food is no longer safe to consume, thereby reducing food waste and ensuring consumer safety^170^.

The most recent developments in intelligent and active packaging technologies, including oxygen scavengers and antimicrobial coatings, are intended to actively interact with food in order to enhance safety and extend the shelf life. Furthermore, multilayer packaging techniques, which integrate various materials to optimize mechanical strength and barrier properties, are guaranteeing superior food preservation^171^.

By preventing gas exchange and moisture ingress, advancements in barrier coatings and film technologies are crucial for the preservation of food quality. Additionally, biodegradable materials for active packaging provide sustainable alternatives to conventional plastics while simultaneously ensuring the preservation of food. Packaging waste is reduced and a sustainable packaging solution is provided by edible packaging solutions that can be ingested in conjunction with the food. The mechanical strength and antimicrobial properties of halloysite nanotubes in packaging materials are enhanced, thereby enhancing food safety and expiration life. The future of sustainable food preservation is contingent upon the ongoing development and adoption of such advanced packaging technologies. These innovations address the environmental impact of packaging waste, extend the shelf life of food, and maintain its integrity^172^.

Innovation in preservation technologies plays a pivotal role in enhancing food longevity, ensuring food security, and minimizing post-harvest losses. Emerging techniques like cold plasma, pulsed light technology, High pressure processing, ultraviolet (UV) radiation, ozone treatment, pulsed electric field, and smart packaging offer promising, eco-friendly solutions that retain the nutritional and sensory qualities of food while extending shelf life. These advancements not only reduce dependence on conventional preservation methods but also support sustainable supply chains by curbing food waste. Moving forward, integrating these technologies with digital tools like IoT and AI can further optimize storage and distribution, making food systems more resilient and efficient in the face of global challenges.

## Global food security challenges and solutions: a 2024 perspective

In 2024, food security continues to be a pressing global challenge; the World Bank has pledged $45 billion to tackle food and nutrition insecurity, a significant increase from $30 billion in 2022. This investment aims to safeguard livelihoods and address the growing disparities in food access. However, these disparities are exacerbated by economic factors such as inflation and extreme weather events. The World Food Security highlights that there is a slow stabilization in food security conditions, particularly in low-income regions, where economic pressures persistently hinder progress. Although the global population is growing and the strain on resources like land, water and energy is increasing, this only complicates the challenges we face. As financing requirements are anticipated to soar to $90 billion per year between 2027 and 2029, there exists a pressing necessity for ongoing investment in social safety nets, malnutrition prevention and the transformation of food systems^173^.Technological advancements (such as plant-based and lab-cultured meats, hydroponics and alternative proteins like microalgae and insects) provide sustainable solutions that can mitigate environmental impact and safeguard food security. Furthermore, improvements in food processing technologies, as well as supply chain efficiency, are crucial for reducing waste, conserving resources and enhancing food accessibility^174^. Although addressing food security is a complex challenge, it necessitates a coordinated global response to adapt to emerging difficulties and ensure a sustainable future for food.

## Future directions to enhance global food security

### Consumer education and cultural integration

In order to effectively incorporate alternative food sources into mainstream diets, it is essential to prioritize public perception and acceptance. Cultural unfamiliarity or aesthetic aversion are frequently the causes of consumer resistance, particularly in the context of insect-based or seaweed-derived products. Culinary innovation, nutrition awareness programs, and public health campaigns can be instrumental in the destigmatization of these innovative foods. The incorporation of these foods into well-known recipes, their inclusion in school meal programs, and their promotion by chefs or influencers can assist in the transformation of consumer attitudes. Furthermore, the encouragement of informed acceptance can be facilitated by providing education on the environmental and nutritional advantages of these sources^175^.

### Policy support and regulation

It is imperative to establish a regulatory framework that is both transparent and robust in order to foster trust among investors and consumers. In order to facilitate alternative food production, governments must prioritize the development of safety standards, labeling norms, and ethical guidelines for lab-grown meat, insect-based products, and genetically modified crops. These regulations not only guarantee product safety but also encourage investment from both the public and private sectors. Cross-border collaboration and trade in the alternative food sector could also be facilitated by international harmonization of food safety regulations^176,177^.

### Technology access for smallholders

In order for global food systems to be sustainable and inclusive, smallholder farmers must have access to technological innovations such as hydroponics, vertical farming, and climate-resilient crop cultivation. This can be accomplished by means of government-led subsidies, public-private partnerships, and localized training programs. Providing rural and marginalized communities with affordable infrastructure and expertise ensures the diversification of food production and enhances their resilience to climate-induced agricultural disruptions^178^.

### Research and Development (R&D)

Enhancing the efficacy and appeal of alternative foods necessitates innovative research. The optimization of sensory properties (taste, texture, aroma), nutritional enhancement, cost reduction, and shelf-life extension of novel foods should be the primary focus of investments. Furthermore, research and development must prioritize sustainable production processes that minimize energy, water, and land consumption. Impactful and scalable solutions can be advanced through interdisciplinary collaboration among food scientists, technologists, environmentalists, and policy experts^179^.

### Circular economy integration

The implementation of circular economy principles in the food production sector has the potential to substantially reduce waste and improve resource efficiency. For example, agricultural byproducts can be utilized as nutrient sources in aquaponic systems or as feed in insect cultivation. The bioconversion of food waste into high-protein biomass, compost, or bioenergy can establish closed-loop systems that generate economic value and promote environmental sustainability. It is imperative to advocate for these practices at the community and industry levels in order to establish resilient agricultural networks^180^.

### Global collaboration

Coordinated international action is necessary to achieve global food security. Institutions such as the FAO, WHO, and World Bank should be instrumental in promoting equitable access to alternative agricultural technologies by facilitating research sharing, funding mechanisms, and policy frameworks. Developing nations can be guaranteed that they are not excluded from the adoption of sophisticated food systems through cross-country collaborations. Initiatives such as food diplomacy, joint ventures, and knowledge exchange platforms can further cultivate a shared vision for sustainable and inclusive food security^180^.

## Future challenges to be circumvented

Utilizing future foods to achieve Sustainable Development Goal 2 (SDG-2) poses a number of difficult challenges that need to be successfully overcome. Although scientific and technical developments have accelerated the development of sustainable, nutrient-dense, and functional food products, significant obstacles still stand in the way of their broad acceptance and influence. The acceptability and impression of consumers is one of the biggest obstacles. The safety, flavor, and nutritional value of innovative foods like lab-grown meat, insect-based proteins, and single-cell proteins are still questioned, despite increased knowledge of sustainable food sources. Strong education, clear labeling, and clever marketing techniques are necessary to break through these psychological and cultural obstacles, gain the trust of consumers, and promote broader adoption^181^.

The frameworks of regulations and policies present a further significant challenge. In order to guarantee food safety, labeling compliance, and ethical considerations, extensive, internationally harmonized rules are required, as future foods frequently use innovative processing methods, bioengineering, and synthetic biology. Regulatory agencies have to quickly adjust to new technology while maintaining environmental sustainability and public health. Another significant issue is the price and scalability of future foods. High production costs, resource-intensive procedures, and supply chain constraints continue to impede large-scale commercialization, despite promising initial research and pilot-scale production. Achieving food security goals will depend on making sure these items are affordable and widely available, especially in low- and middle-income nations.

The trajectory of future food uptake is further complicated by worries about environmental sustainability. Despite the fact that many alternative food sources seek to lessen land exploitation, water consumption, and carbon footprints, some methods—like cellular agriculture and precise fermentation—still demand significant energy inputs. Reducing the environmental impact of future foods will require incorporating renewable energy sources and energy-efficient technologies into food production systems. Furthermore, food safety and nutritional optimization continue to be crucial. Even if a lot of upcoming foods promise improved nutritional profiles, problems including allergenicity, anti-nutritional variables, and long-term health impacts need careful research. Further investigation on digestibility, bioavailability, and other health hazards will be necessary to guarantee that future foods improve human health without causing unexpected side effects^182^.

Finally, socio-economic and ethical considerations must not be overlooked. Traditional agriculture-based livelihoods may be disrupted by the shift to future food systems, requiring equitable policies and workforce reskilling to help impacted people. To promote acceptance and responsible innovation, ethical discussions over genetic changes, lab-grown foods, and biotechnological interventions need to be addressed with precise rules and public involvement. Policymakers, scientists, industry stakeholders, and consumers must work together in a multidisciplinary manner to address these issues. The future of food can genuinely correspond with the goals of SDG-2 by proactively removing these obstacles, guaranteeing a sustainable, wholesome, and equitable food system for future generations.

## Conclusion

The study underscores the urgent need for a resilient and sustainable food system to address the challenges posed by population growth, limited resources, economic pressures, and shifting food habits. By 2050, innovative approaches such as alternate food sources, including edible insects, seaweeds, plant-based and lab-cultured meats, and underutilized crops, along with advancements in hydroponics and next-generation fish farming, will play a pivotal role in transforming food systems. Technological interventions, including artificial intelligence (AI), blockchain, biotechnology, and robotics, are set to revolutionize agriculture and food processing by enhancing decision-making, optimizing resource use, predicting crop yields, automating supply chains, and minimizing food loss. AI-driven precision farming, smart sensors, and predictive analytics enable real-time monitoring and adaptive responses to environmental and market conditions, further boosting sustainability. Consumer acceptance of engineered foods and fortified products will be key to bridging the gap between innovation and implementation. Collectively, these strategies offer a pathway to a secure, transparent, and sustainable food future for the global population.

## Data Availability

No datasets were generated or analysed during the current study.
